# FKBP51 plays an essential role in Akt ubiquitination that requires Hsp90 and PHLPP

**DOI:** 10.1038/s41419-023-05629-y

**Published:** 2023-02-13

**Authors:** Martina Tufano, Laura Marrone, Chiara D’Ambrosio, Valeria Di Giacomo, Simona Urzini, Yichuan Xiao, Monica Matuozzo, Andrea Scaloni, Maria Fiammetta Romano, Simona Romano

**Affiliations:** 1 Department of Molecular Medicine and Medical Biotechnology, University of Naples Federico II, 80131 Naples, Italy; 2 Proteomics, Metabolomics and Mass Spectrometry Laboratory Institute for Animal Production Systems in Mediterranean Environments (ISPAAM), National Research Council (CNR), Piazzale Enrico Fermi 1, Portici, 80055 Naples, Italy; 3 Chinese Academy of Sciences Key Laboratory of Tissue Microenvironment and Tumor, Shanghai Institute of Nutrition and Health, University of Chinese Academy of Sciences, Chinese Academy of Sciences, Shanghai, China

**Keywords:** Ubiquitylation, Melanoma

## Abstract

FKBP51 plays a relevant role in sustaining cancer cells, particularly melanoma. This cochaperone participates in several signaling pathways. FKBP51 forms a complex with Akt and PHLPP, which is reported to dephosphorylate Akt. Given the recent discovery of a spliced FKBP51 isoform, in this paper, we interrogate the canonical and spliced isoforms in regulation of Akt activation. We show that the TPR domain of FKBP51 mediates Akt ubiquitination at K63, which is an essential step for Akt activation. The spliced FKBP51, lacking such domain, cannot link K63-Ub residues to Akt. Unexpectedly, PHLPP silencing does not foster phosphorylation of Akt, and its overexpression even induces phosphorylation of Akt. PHLPP stabilizes levels of E3-ubiquitin ligase TRAF6 and supports K63-ubiquitination of Akt. The interactome profile of FKBP51 from melanoma cells highlights a relevant role for PHLPP in improving oncogenic hallmarks, particularly, cell proliferation.

## Introduction

Protein kinase B (PKB/Akt) is a member of the serine-threonine kinase AGC superfamily that is often constitutively active in tumors and assumes a relevant role in cancer growth and resistance [1–3]. Akt is activated through the binding of phosphatidylinositol 3,4,5-triphosphate (PIP3) to its N-terminal PH domain, which affects the structure of Akt and recruits it to the plasma membrane, where phosphoinositide-dependent kinase 1 (PDK1) phosphorylates the protein activation loop at threonine 308 (T308) [4]. Phosphorylation at T308 promotes a protein conformational change that enables Akt to bind to ATP, thus allowing phosphate transfer [5]. Further phosphorylation of the hydrophobic motif at serine 473 (S473) occurs by the mammalian/mechanistic target of rapamycin complex 2 (mTORC2) [6]. Phosphorylation in Akt can also occur at threonine 450, but this modification takes place constitutively during translation and is important for kinase stability [7]. Upon phosphorylation, phosphorylated Akt (pAkt) is detached from the membrane and translocates to the target sites in the cytoplasm and nucleus. Different phosphatases tightly control the Akt pathway; in particular, protein phosphatase A (PP2A) dephosphorylates T308, whereas PH domain leucine-rich repeat protein phosphatase (PHLPP) dephosphorylates S473 [8]. Both events induce the inactivation of Akt, switching off kinase pro-survival and growth-promoting signaling [8].

Among the known Akt interactors, the immunophilin FKBP51 acts as a scaffold protein forming a complex with Akt and PHLPP [9, 10]. FKBP51 is a large immunophilin encoded by the *FKBP5* gene, localized on the short arm of chromosome 6 (6p21.31) [11]. The protein contains two N-terminal FKBP-like domains, FK1 and FK2, separated by a short linker sequence [11]. Only the FK1 domain exerts the PPIase and ligand binding activity, while FK2 is inactive, but it contains an ATP/GTP-binding sequence and seems to retain an interaction ability and a structural role [12]. FKBP51 mainly functions as a scaffold [13]. FKBP51 has been shown to exert isomerase activity on IKK kinase complex [14], and the human telomerase reverse transcriptase (hTERT) [15]. The C-terminal region has a TPR domain involved in interacting with other proteins, particularly the chaperone Hsp90 [16]. By alternative splicing events, the *FKBP5* gene generates a truncated isoform (FKBP51s) lacking the TPR domain because of a frameshift yielding an early stop-codon and, consequentially, a non-conventional C-terminal sequence [17].

Pei and coworkers originally discovered the complex FKBP51/Akt/PHLPP and proposed a role for FKBP51 as a scaffold protein that, by bringing together Akt and its phosphatase, enhanced the de-phosphorylation of this kinase in a pancreatic cancer context [9]. They found that the deletion of either FK1 or FK2 domains in FKBP51 abolished the interaction of the protein with Akt, while its TPR domain was involved in the binding to PHLPP [9]. Conflicting studies showed that FKBP51 upregulated Akt activation in cancer [10, 18]. In a mouse model of melanoma, we found that treatment with FKBP51siRNA decreased Akt phosphorylation at S473 in both normal (locoregional lymph nodes) and cancerous tissues (xenografts) [18]. Conversely, Fabian and coworkers observed an increase in Akt phosphorylation at S473 upon FKBP51 overexpression. The latter investigators showed that Akt could directly bind to FKBP51 via the FK1 domain or, indirectly, via the TPR domain through Hsp90 [10]. Moreover, they proposed that the FKBP51-Akt interaction was sensitive to Akt conformation [10]. More recently, the research group that first identified the role of FKBP51 as a scaffold protein for PHLPP reported that this chaperone does not necessarily assist the de-phosphorylation of Akt. Still, they showed that this capability depends on the acetylation status of the immunophilin [19].

In 2014, Akiyama and coworkers demonstrated an interaction between tumor necrosis receptor-associated factor (TRAF) family members and FKBP51 [20]. They identified a N-terminal FK1 flanking sequence (aa 13-30) of FKBP51 as a putative binding site for TRAF6 and demonstrated the latter binding to this immunophilin through its C-terminal domain [20]. TRAF6 is one of the best-studied E3 ligases of Akt [21, 22]. It is a cytoplasmatic adaptor protein that belongs to the tumor necrosis factor receptor (TNFR) superfamily together with five other canonical members (TRAF1-5) and one non-canonical member (TRAF7). The N-terminal of TRAF6 contains a RING domain with E3 ubiquitin ligase activity and five zinc fingers that provide structural support [23].

The ubiquitination system exerts a pivotal role in the control of the Akt turnover and activation. It promotes plasma membrane and/or nuclear translocation, thus facilitating the cell-surface-receptor turnover and the control of gene transcription [24]. The Akt kinase activity can be modified by both lysine 48 (K48)- and lysine 63 (K63)-associated protein polyubiquitination [24]. K48-linked ubiquitination is thought to control proteasomal degradation of Akt, thereby effectively shutting off Akt activity and corresponding downstream responses [24]. In contrast, K63-linked polyubiquitination is thought to serve as a regulatory signal that promotes localization, trafficking and plasma membrane recruitment of Akt [25]. The diversity of the biological actions that can be controlled by ubiquitination/deubiquitination is due to both the high substrate specificity of the ubiquitin system and the variety of regulatory mechanisms and different protein effectors controlling ligases and deubiquitinases [26]. TRAF6 is among the reported players involved in Akt K63-linked polyubiquitination, which also includes E3 ligase S-phase kinase-associated protein 2 (Skp2) that mediates epidermal growth factor (EGF) signaling via Akt [27], and the neuronal precursor cell-expressed developmentally downregulated 4 (NEDD4) that positively regulates the nuclear trafficking of the activated Akt [28].

In this study, we interrogated the two FKBP5 isoforms in the regulation of Akt activation and the underlying regulatory mechanisms. We hypothesized that the *FKBP5* gene, through alternative splicing, may modulate phosphorylation and de-phosphorylation of Akt, reconciling the diverging results from the literature. Supported by the notion of FKBP51 binding to TRAF6 [20], we hypothesized and investigated the involvement of the canonical protein in Akt ubiquitination/phosphorylation and the spliced isoform action as a dominant-negative, being unable to support Akt ubiquitination.

## Materials and methods

### Cell culture and reagents

We used A375 and A2058 human melanoma cell lines, and D54MG and U251MG glioblastoma (GBM) cell lines, kindly provided by CEINGE cell bank (Cellular Technology Platform, https://www.ceinge.unina.it/en/cell-cultures) at the Advanced Biotechnology Institute (Naples, Italy). Cell line A375 [29] was cultured in Dulbecco’s Modified Eagle’s Medium (DMEM) (Corning, Glendale, Arizona, USA) supplemented with 15% heat-inactivated fetal bovine serum (FBS)(Corning), 200 mM glutamine (Corning), and 100 U/ml penicillin-streptomycin (Biowest, Nuaillé, France). Cell line A2058 [30], glioblastoma primary cell line GB138 established from acutely resected human GBM [31] and HEK293 human kidney cell line [32] were cultured in DMEM (Corning) supplemented with 10% heat-inactivated FBS (Corning, Glendale, Arizona, USA), 200 mM glutamine (Corning) and 100 U/ml penicillin-streptomycin (Biowest). D54MG [33], U251MG [34] were cultured in DMEM/Hams F-12 50/50 (Corning) supplemented with 10% heat-inactivated FBS (Corning), 200 mM glutamine (Corning) and 100 U/ml penicillin-streptomycin (Biowest). SU86 were purchased by ATCC (ATCC-CRL-1837) and cultured with RPMI 1640 (Corning) supplemented with 10% heat-inactivated FBS (Corning, Glendale, Arizona, USA), 200 mM glutamine (Corning) and 100 U/ml penicillin-streptomycin (Biowest). All the cell lines were kept at 37 °C in a 5% CO_2_ humidified atmosphere and were mycoplasma free.

A375 cells stably knocked down with an FKBP51 short hairpin RNA (Sh FKBP51) or the control cells stably transfected with shRNA (Sh Ctrl) were obtained as previously described [14]. For the establishment of A375, A2058, and GB138 knockout cell lines, cells were transfected with a CRISPR/Cas9 KO plasmid along with a homology-directed-repair (HDR) plasmid for the puromycin resistance (Santa Cruz Biotechnology, Dallas, Texas, USA). Control cells were obtained transfecting HDR plasmid alone. After 24 h from transfection, cells were selected with 200 ng/ml puromycin (Merck, Darmstadt, Germany). Transfected cells that survived to puromycin were further seeded with the limiting dilution technique in order to generate single FKBP51-KO clones [35].

For the experimental procedures, 17-(allylamino)-17-demethoxygeldanamycin (17-AAG) (Merck KGaA, Darmstadt, Germany) was diluted in DMSO to make a stock solution of 10 mg/ml, in accordance with the manufacturer’s recommendations. Treatment with 0.5–1 µM 17-AAG was performed 16 h before cells were collected.

### Plasmids, short interfering oligoribonucleotides and transfection

For overexpression and silencing experiments, cells were seeded in 6-multiwell plates at concentration of 4 × 10^5^ per well to obtain confluency of 60–70%. After 24 h, cells were transfected with Metafectene Transfection System (Biontex, Munich, Germany) in accordance with the manufacturer’s recommendations; 3 μg of plasmid of interest were transfected per each well, and cells were harvested after 36 h from transfection. The pcDNA expressing vector encoding hemagglutinin (HA)-tagged human Akt1 was a gift of Prof. Gerolama Condorelli (University of Naples Federico II). Plasmids pRK5 3xFlag-tagged empty vector (EV), pRK5-HA-tagged K63-Ub, and pCLXSN-HA-tagged K63R-Ub [36] were kindly provided by Prof. Ted Dawson (The Johns Hopkins University). PcDNA3 HA-tagged TRAF6 was a gift of Prof. Shao-Cong Sun (MD Anderson Cancer Center, Houston, TX, USA), while PcDNA3 HA-tagged PHLPP1 full length was purchased from Addgene (#37100) [37]. True-ORF-Myc-DDK (Flag)-tagged human FKBP51-transcript variant 1 (canonical FKBP51) and FKBP51-transcript variant 4 (FKBP51s) were purchased from OriGene Technologies (MD, USA) [17]; plasmids encoding for mutants of FKBP51 were pRK5 Flag-tagged FKBP51-TPRmut (K352A/R356A) and pRK5 Flag-tagged FKBP51-PPIasemut (FD67DV), which were kindly provided by Prof. Theo Rein (Max Planck Institute of Psychiatry, Munich, Germany) [38]. Transient knockdown was performed using specific short interfering (siRNA) oligoribonucleotides from QIAGEN (Valencia, California, United States) at a final concentration of 50 nM; No Sense RNA (NS RNA, QIAGEN) was used as control. Validated siRNAs were used for FKBP51 silencing (NM_001145775, #SI02780372 Hs FKBP5_5), TRAF6 silencing (a mix of 4 siRNAs: #SI03050145, #SI03050143, #SI00066122, #SI0006601), and PHLPP silencing (a mix of 4 siRNAs: 2 directed against PHLPP1, NM_194449, #SI05126877 Hs_PHLPP_6, #SI04256427 Hs_PHLPP_5; 2 against PHLPP2, NM_015020, #SI04210843 Hs_PHLPP_4, #SI05043773 Hs_PHLPP_7). For FKBP51s silencing, a mix of 3 siRNAs made of siRNA #1 and #2 targeting the 3′-coding region (between 700 and 1100 bp), and siRNA #3 targeting the 3′-UTR region (between 5200 and 5800 bp), was used as described previously [39], which was custom produced by QIAGEN.

### Immunoblotting and antibodies

To obtain whole lysates, cells were collected, and cellular pellets were homogenized in modified RIPA buffer (50 mM Tris-HCl pH 7.5, 125 mM NaCl, 1% NP-40, 0.25% Na-deoxycholate, 1 mM Na-fluoride, 1 mM Na-orthovanadate, 1 mM phenylmethanesulfonylfluoride (PMSF), 1 mM dithiothreitol (DTT), protease inhibitor cocktail) [40]. After 30 min of incubation on ice, lysates were centrifuged ad 14.000 rpm for 15 min to remove cell debris and the supernatant was saved for immunoblotting (IB) assay. Protein concentration was determined using the Bradford protein assay (Bio-rad, Hercules, CA, USA), measuring absorbance at 595 nm. Cell lysates were equalized for total proteins, and the final volume was levelled with water and Laemmli sample buffer (LB). Samples were denatured for 5 min at 95 °C, then loaded in 8/10% T SDS-PAGE and transferred onto a methanol-activated PVDF membrane (Immobilon-P, Millipore, Darmstadt, Germany). The membranes were incubated with the corresponding primary antibody, at 4 °C, overnight. Primary antibodies against the following proteins were diluted as follows: M2-Flag (clone M2, F3165, mouse monoclonal, Sigma-Aldrich, St. Louis, Missouri, USA), γ-tubulin (clone DM1A, T9026, mouse monoclonal, Sigma-Aldrich) and PHLPP (22789-1-AP, rabbit polyclonal, ProteinTech, Manchester, UK) were used at a dilution of 1:5000. FKBP51 (NB100-68240, rabbit polyclonal, Novus Biological, Abingdon, UK), FKBP51 (clone Hi51B, ab79844, mouse monoclonal, Abcam, Cambridge, UK) and vinculin (clone G-11, sc-55465, mouse monoclonal, Santa Cruz Biotechnology) were used at a dilution of 1:3000. HA (clone GT423, GTX 628489, mouse monoclonal, GeneTex, Irvine, California, USA) was used at a dilution of 1:2000. PAkt s473 (clone D9E, #4060, rabbit monoclonal), p-p70S6K (rabbit polyclonal, #9205), Cyclin D1 (#2922 rabbit polyclonal), K63 polyubiquitin (clone D7A11, #5621), K48 polyubiquitin (#4289, rabbit polyclonal, Cell Signaling, Danvers, Massachusetts, USA), GM-130 (clone D6B1, #12480, rabbit monoclonal, Cell signaling, Danvers, Massachusetts, USA), TRAF6 (A16991, rabbit polyclonal, Abclonal, Woburn, Massachusetts, USA), G3PDH (clone 6C5, sc-32233, mouse monoclonal, Santa Cruz Biotechnology), PI4K2B (15074-1-AP, rabbit polyclonal, ProteinTech Manchester, UK) and FAM84B (E-AB-17945, rabbit polyclonal, Elabscience Houston, Texas, USA) were used 1:1000. Akt1/2/3 (clone 5C10, sc-81434, rabbit polyclonal, Santa Cruz Biotechnology), Hsp90 α/β (clone F-8, sc-13119), p70S6k (clone H-9, sc-8418), TRAF6 (clone D-10, sc-8409) and RAB11A (clone A-6, sc-166912, mouse monoclonal, Santa Cruz Biotechnology) and FKBP51s (rabbit polyclonal home-made) [16] were used at a dilution of 1:500. After washes, membranes were incubated with secondary antibodies for 1 h, at room temperature. Anti-mouse and anti-rabbit secondary antibodies HRP-conjugated were purchased from ImmunoReagents (Raleigh, Carolina del Nord, USA) and diluted 1:5000. IBs were revealed with Western Blotting Luminol Reagent (Santa Cruz Biotechnology). A quantification of bands was obtained by densitometry analysis using ImageJ 1.42q for Macintosh; integrated optical densities (ODs) of each analyzed protein were normalized to a relative housekeeping gene, and values were expressed as fold change of protein levels in the different samples in comparison with a control sample whose expression was arbitrarily indicated equal to 1 [41]. We assessed the linearity of our detection system in accordance with McDonough et al. [42].

### Immunoprecipitation and ubiquitination assay

Cells were lysed in modified RIPA buffer and subjected to immunoprecipitation (IP) as previously described [42]. Briefly, 500 µg of whole lysates were incubated with the primary antibody (1 µg/mL), at 4 °C, under rotation, or with IgG (control); 50 µg of the whole lysates was saved as input fraction for further analysis. After 16 h, 25 µL (for each point) of Protein-A/G-coupled agarose beads (Santa Cruz Biotechnology) were added to each mixture, which was further incubated for 2 h, at 4 °C, under rotation. Then, resin-associated proteins were washed 3 times with standard lysis buffer, eluted with LB buffer and separated by SDS–PAGE, along with the input fraction, as above described. For the in vivo ubiquitination assay, cells were lysed in modified RIPA buffer as previously described [43]. After saving 10% of the whole lysates as input for further analysis, the remaining cell extracts were boiled in the presence of 1% w/v SDS, for 5 min, and then 10-fold diluted with RIPA buffer. Diluted lysates were pre-cleaned with Protein-A/G-coupled agarose beads, and then incubated with specific immunoprecipitation antibodies at 4 °C, overnight, on a shaker. Then, immunoprecipitated proteins were collected by incubation with Protein-A/G-coupled agarose beads at 4 °C, for 2 h, on a shaker, washed with RIPA buffer containing protease inhibitors, PMSF and N-ethylmaleimide, boiled at 100 °C for 5 min, and then loaded for further SDS-PAGE analysis. Resolved proteins were immunoblotted with anti-K63 ubiquitin or the indicated antibodies. For the in vitro ubiquitination assay, 7 × 10^6^ HEK293 cells were seeded in 15 cm-dishes and, after 24 h, were transfected as described above to obtain the ectopic expression of HA-Akt, HA-TRAF6 along with, or without, the over-representation of FKBP51. After 24 h, transfected cells were lysed and about 9 mg of proteins were immunoprecipitated with 15 μL monoclonal Anti-HA Agarose (Merck KGaA), while the remaining 0.9 mg of cell lysates were used as input fractions for further analysis. Diluted lysates were pre-cleaned with Protein-A/G-coupled agarose beads, incubated with the monoclonal Anti-HA Agarose at 4 °C, for 3 h, on a shaker, and, then assayed for in vitro ubiquitination. In vitro ubiquitination reactions were performed with the Ubiquitination kit from Boston Biochem (Cambridge, MA, USA), according to the manufacturer’s instructions and as previously described [44]. The reactions were terminated by boiling samples in SDS-PAGE sample buffer for 5 min, and were subjected to electrophoresis followed by immunoblot analysis to examine the ubiquitination of Akt.

### Proteomic analysis

For proteomic analysis, 15 × 10^6^ A375 cells were seeded in 15 cm-dishes; after 24 h, they were transfected as described above to obtain the ectopic expression of FKBP51 along with, or without, the over-representation of PHLPP. As control cells, we used A375 cells transfected with an empty vector (EV). After 36 h, transfected cells were harvested and 90% of the corresponding whole lysates were immunoprecipitated with 600 μL of PierceTM Anti-DYKDDDDK Magnetic Agarose (Invitrogen Waltham, MA, USA), while the remaining 10% ones were used as input fractions for further analysis. Pre-cleaning of the resin was performed in accordance with the manufacturer’s recommendations. Hence, samples were probed with the resin and incubated on a shaker, at 37 °C, for 20 min. Then, loaded resin samples were washed three times with PBS, and then eluted with 100 μL of LB buffer. Immunopurified protein samples were analyzed in parallel by 8% T SDS-PAGE. After staining with colloidal Coomassie blue, whole gel lanes were cut into 15 slices, which were minced and washed with water. Corresponding proteins were separately in gel reduced, S-alkylated with iodoacetamide and digested with trypsin, as previously reported [45].

Individual protein digests were then analyzed with a nanoLC-ESI-Q-Orbitrap-MS/MS platform consisting of an UltiMate 3000 HPLC RSLC nano system (Thermo Fisher Scientific, USA) coupled to a Q-ExactivePlus mass spectrometer through a Nanoflex ion source (Thermo Fisher Scientific). Peptides were loaded on an Acclaim PepMapTM RSLC C18 column (150 mm × 75 μm ID, 2 μm particles, 100 Å pore size) (Thermo Fisher Scientific), and eluted with a gradient of solvent B (19.92/80/0.08 v/v/v water/acetonitrile/formic acid) in solvent A (99.9/0.1 v/v water/formic acid), at a flow rate of 300 nl/min. The gradient of solvent B started at 3%, increased to 40% over 40 min, raised to 80% over 5 min, remained at 80% for 4 min, and finally returned to 3% in 1 min, with a column equilibrating step of 30 min before the subsequent chromatographic run. The mass spectrometer operated in data-dependent mode using a full scan (*m/z* range 375–1500, a nominal resolution of 70,000, an automatic gain control target of 3,000,000, and a maximum ion target of 50 ms), followed by MS/MS scans of the 10 most abundant ions. MS/MS spectra were acquired in a scan *m/z* range 200–2000, using a normalized collision energy of 32%, an automatic gain control target of 100,000, a maximum ion target of 100 ms, and a resolution of 17,500. A dynamic exclusion value of 30 s was also used. Duplicate analysis of each sample was performed to increase the number of identified peptides/protein coverage.

MS and MS/MS raw data files per lane were merged for protein identification into Proteome Discoverer v. 2.4 software (Thermo Fisher Scientific), enabling the database search by Mascot algorithm v. 2.6.1 (Matrix Science, UK) with the following parameters: UniProtKB human protein database (214889 sequences, 02/2020) including the most common protein contaminants; carbamidomethylation of Cys as fixed modification; oxidation of Met, deamidation of Asn and Gln, and pyroglutamate formation of Gln as variable modifications. Peptide mass tolerance and fragment mass tolerance were set to ±10 ppm and ±0.05 Da, respectively. Proteolytic enzyme and maximum number of missed cleavages were set to trypsin and 2, respectively. Protein candidates assigned on the basis of at least two sequenced peptides and Mascot score ≥30 were considered confidently identified. Definitive peptide assignment was always associated with manual spectra visualization and verification. Results were filtered to 1% false discovery rate. A comparison with results from the corresponding control (EV) allowed to identify contaminant proteins that, nonetheless their abundance, were removed from the list of putative FKBP51-interacting partners, yielding the inventory reported in Supplementary Information, Table S1. Data are available via ProteomeXchange with identifier PXD033828.

Functional enrichment analysis was performed by using FunRich 3.1.3 (http://www.funrich.org). STRING (https://string-db.org) was used to visualize and integrate complex networks of experimental data.

## Results

### FKBP51 and FKBP51s differentially act on Akt phosphorylation

**Fig. 1:**
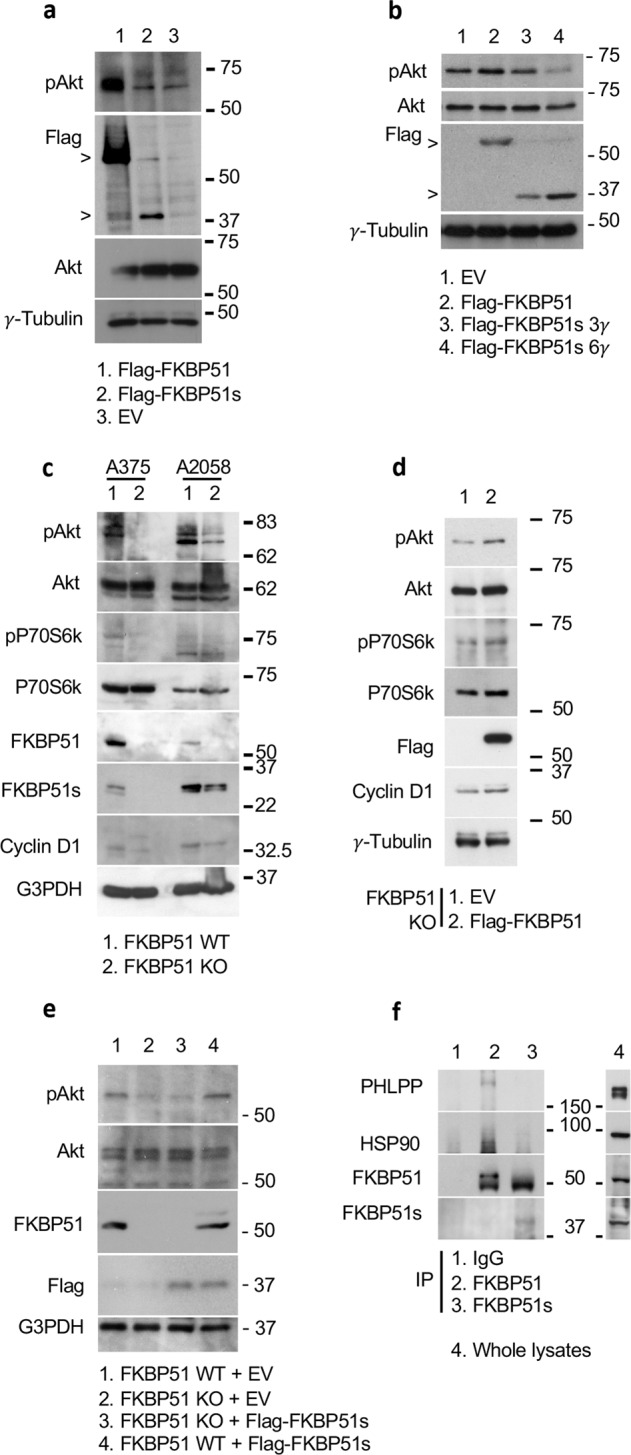
FKBP51 and FKBP51s effect on Akt phosphorylation in melanoma. **a** IB of A375 cell transfected with Flag-FKBP51, Flag-FKBP51s or correspondent EV as control. The canonical FKBP51, but not the spliced FKBP51, increased pAkt levels. **b** IB of A375 cell transfected with Flag-FKBP51 or increasing amounts (3 and 6 μg) of Flag-FKBP51s. PAkt levels decreased in FKBP51s overexpressing cells in a dose-dependent manner. **c** IB assay of A375 and A2058 melanoma cell lines stably knocked out for FKBP51 with the CRISPR/Cas9 technology. Compared to control (WT) cells, KO cells assay showed impaired levels of pAkt and its downstream targets. **d** IB of pAkt, p-P70S6k and cyclin D1 levels in A375 KO cells rescued or not with Flag-FKBP51. Activation of Akt pathway was restored upon FKBP51 rescue. P-P70S6k, P70S6k and cyclin D1 were analyzed on a blot run in parallel; note that in the second blot, some samples were not loaded due to limiting quantities (Supplementary information). **e** IB of pAkt levels in WT and KO cells overexpressing Flag-FKBP51s. FKBP51s did not restore pAkt levels in A375 KO cells. FKBP51 was analyzed on a blot run in parallel (Supplementary information). **f** IP assay of A375 melanoma cells. Endogenous FKBP51 or FKBP51s were immunoprecipitated with anti-FKBP51 or anti-FKBP51s antibodies, while IgG served as control for a non-specific binding. IB showed that PHLPP interacted only with the canonical FKBP51 isoform. IB of the whole lysate is also shown.

We overexpressed FKBP51 and FKBP51s in A375 melanoma cells, and measured pAkt levels by IB. As shown in Fig. 1a, pAkt was increased following FKBP51 overexpression, in comparison with EV-transfected cells. Conversely, pAkt levels in FKBP51s-overexpressing cells remained comparable to those of the control. Interestingly, when we doubled concentration of Flag-FKBP51s, pAkt levels almost disappeared (Fig. 1b). Through the CRISPR/Cas9 technology, we then generated two FKBP51 KO cell lines using A375 and A2058 melanoma cell lines as starting materials. These FKBP51 KO cells and corresponding controls were assayed by IB for pAkt and its downstream targets P70S6 kinase (P70S6k) and cyclin D1. IB showed impaired activation of the Akt pathway in both KO cells (Fig. 1c). FKBP51 rescue of A375 KO restored Akt activation (Fig. 1d). Overexpression of FKBP51s in KO clone did not reactivate Akt phosphorylation (Fig. 1e). Quantification of bands from each blot is shown in Supplementary Information (Fig. S1a–e). By using an A375 cell line stably downmodulated for FKBP51 by a short hairpin RNA (ShFKBP51) and corresponding control cells transfected with a non-silencing Sh (ShCtrl) [14], we further showed that pAkt levels are reduced in ShFKBP51- in comparison to ShCtrl-treated cells (Supplementary Information, Fig. S2a). Ectopic FKBP51s reduced Akt phosphorylation (Supplementary Information, Fig. S2a). The supporting role of FKBP51 but not FKBP51s in Akt phosphorylation was confirmed in a different neoplasia, namely glioblastoma, constitutively expressing high levels of both FKBP51 isoforms [39, 46]. FKBP51 but not FKBP51s silencing in D54 cell line hampered pAkt levels (Supplementary Fig. S2b). Moreover, two FKBP51 KO clones generated by CRISPR/Cas9 in GB138 cell line showed impaired pAkt levels (Supplementary Fig. S2c), when compared with control cells. Altogether, these results suggest that the spliced and not the canonical FKBP51 isoform can deactivate Akt. Because PHLPP was found as responsible for Akt deactivation [9], we looked at PHLPP/FKBP51s interaction. To this end, we immunoprecipitated endogenous FKBP51s and FKBP51 from A375 cells (Fig. 1f) and analyzed the corresponding binding to PHLPP. Our results confirmed PHLPP binding to FKBP51 [9] and showed that FKBP51s is unable to bind PHLPP (Fig. 1f). Similar results were obtained in two different glioblastoma cell lines (Supplementary Information, Fig. S2d). These findings excluded a role for PHLPP in FKBP51s-mediated dephosphorylation of Akt.

### The TPR domain of FKBP51 promotes K63-ubiquitination of Akt

**Fig. 2:**
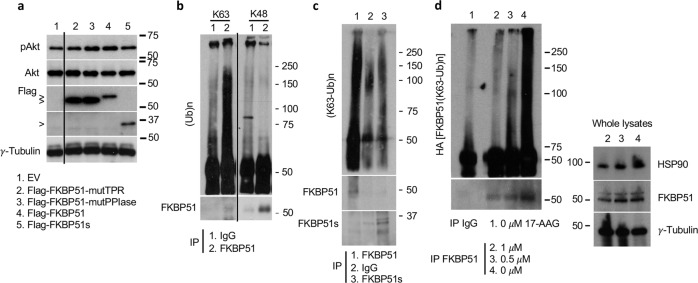
The TPR domain of FKBP51 binds to non-degradative Ub. **a** A375 cells were transfected with FKBP51 forms harboring mutations at protein C-terminus (Flag-FKBP51-mutTPR) or N-terminus (Flag-FKBP51-mutPPIase), Flag-FKBP51, Flag-FKBP51s or correspondent EV as control. IB analysis showed that mutTPR- and FKBP51s-overexpressing cells had reduced pAkt levels, when compared with counterparts overexpressing FKBP51 or mutPPIase. **b** IP assay of endogenous FKBP51 in A375 melanoma cells. IgG served as control for a non-specific binding. Immunoprecipitated proteins were then assayed by IB; the use of specific anti-ubiquitin (K63 or K48) antibodies revealed that FKBP51 binds only to K63-Ub but not to K48 residues. IB of whole lysates is also shown. Note that to optimize the visualization of the FKBP51 band, the membrane was cut (Supplementary information). **c** Endogenous FKBP51 and FKBP51s were immunoprecipitated from whole lysates obtained from A375 melanoma cells. IB showed that the spliced isoform FKBP51s was not bound to K63-Ub residues. FKBP51s was analyzed on a blot run in parallel because a different acrylamide concentration allowed a better resolution of the band; note that in the second blot, also a positive control was loaded (Supplementary information). **d** A375 melanoma cells were treated with 0.5 and 1 μM of the Hsp90 inhibitor 17-AAG. After 16 h, endogenous FKBP51 was immunoprecipitated and anti-K63-Ub antibody served to detect K63-Ub residues. IB showed that FKBP51 binding to K63-Ub was Hsp90-dependent. IB of whole lysates is also shown. γ-Tubulin was used as loading control.

As FKBP51s lacks the TPR, we hypothesized that FKBP51 exploited this domain to induce Akt activation. To address such a hypothesis, we used a FKBP51-TPR mutated protein construct [38] and investigated its effect on pAkt levels. For comparison, we used FKBP51-WT, mutPPIase (lacking the enzymatic function) [38] and FKBP51s. As shown in Fig. 2a, similarly to FKBP51s, mutTPR, but not mutPPIase, reduced pAkt levels in comparison to FKBP51-WT cells. As expected, levels of active P70S6k (Supplementary Information, Fig. S3a) and cyclin D1 (Supplementary Information, Fig. S3b) appeared to be reduced in mutTPR, compared to mutPPIase. Based on the observation that the integrity of TPR is essential for FKBP51 regulation of Akt phosphorylation, we then investigated the action of TPR on the leading mechanism of Akt activation, namely K63-linked ubiquitination [21]. To this end, we first looked at whether FKBP51 could bind K63-Ub, using K48-Ub for comparison. Figure 2b shows that FKBP51 specifically bound to K63-Ub, but not to K48-Ub; thus, FKBP51 binding to K63-Ub appeared to be associated with a non-degradative polyubiquitination. Conversely, FKBP51s was barely bound to K63-Ub, which was consistent with the lack of TPR domain (Fig. 2c).

**Fig. 3:**
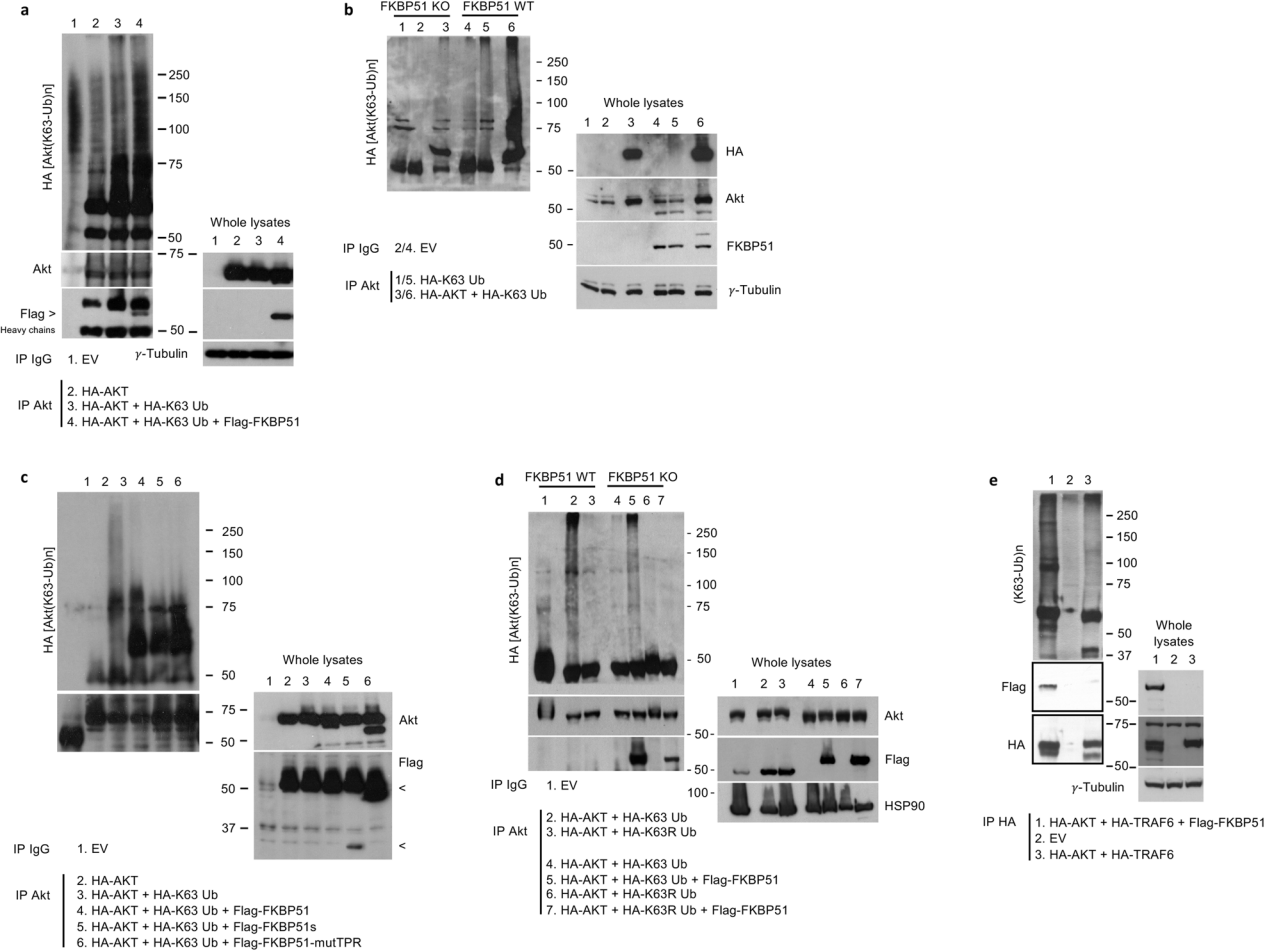
FKBP51 promotes the K63-ubiquitination of Akt in melanoma cells through its TPR domain. **a** IP assay of A375 melanoma cells transfected with HA-Akt, HA-K63-Ub and Flag-FKBP51. Immunoprecipitated protein was then assayed by IB. Anti-HA antibody revealed an increased K63-linked ubiquitination of Akt upon FKBP51 overexpression. IB of whole lysates is also shown; γ-tubulin was used as loading control. **b** A375 melanoma WT and KO for FKBP51 were transfected with HA-Akt and HA-K63-Ub. Then, Akt was immunoprecipitated and assayed with anti-HA. IB assay showed an impaired Akt K63-ubiquitination in KO cells compared to WT cells. IB of whole lysates is also shown; γ-tubulin was used as loading control. **c** IP assay of A375 melanoma cells transfected with combinations of the following expressing vectors: HA-Akt, HA-K63-Ub, Flag-FKBP51, Flag-FKBP51s and Flag-mutTPR. IB assay with anti-HA revealed the K63-Ub residues bind to Akt, with exception of cells overexpressing FKBP51s or the protein mutant at the TPR domain. IB of whole lysates is also shown. **d** A375 WT and KO for FKBP51 were transfected with HA-Akt, HA-K63-Ub and HA-K63R-Ub. FKBP51 was rescued in KO cells, and Akt was then immunoprecipitated and assayed by IB with anti-HA. IB assay showed that FKBP51 overexpression restored Akt K63-ubiquitination only in presence of WT K63-Ub and not with mutated K63R-Ub. IB of whole lysates is also shown, with Hsp90 used as loading control. In all experiments, Akt was immunoprecipitated with anti-Akt antibody and IgG served as control for a non-specific binding. **e** In vitro ubiquitination assay with HA-IP from A375 melanoma cells previously transfected with HA-AKT + HA-TRAF6 + Flag-FKBP51 (lane 1), HA-AKT + HA-TRAF6 (lane 3) or EV (lane 2). The ubiquitination test was performed in presence of Ubc13/Uev1a as ubiquitin-conjugating (E2) enzymes. These proteins were added to ubiquitination reactions consisting of E1, ATP and Ub, as described in the experimental section. Ubiquitinated proteins were detected by IB with anti-ubiquitin antibody.

Because mutTPR cannot bind to Hsp90 [38] and the chaperone plays a relevant role in sustaining pAkt levels [46], we investigated whether FKBP51 binding to K63-Ub requires Hsp90. To this aim, we treated melanoma cells with 0.5 and 1 μM 17-(allylamino)-17-demethoxygeldanamycin (17-AAG), which is a Hsp90 inhibitor. We observed that FKBP51 binding to K63-Ub was reduced in a dose-dependent manner by 17-AAG (Fig. 2d). These results suggested that TPR binding to K63-Ub requires Hsp90. Relative quantification of bands from Fig. 2 blots is shown (Supplementary Information, Fig. S4). We then investigated Akt ubiquitination. To this end, we immunoprecipitated Akt in condition of FKBP51 overexpression (Fig. 3a) or KO (Fig. 3b). FKBP51 increased K63 ubiquitination of Akt in two different melanoma cell lines (Fig. 3a and Supplementary Information, Fig. S5). FKBP51 KO cells showed an impaired binding of Akt to K63-Ub residues (Fig. 3b). In FKBP51s- or mutTPR-overexpressing cells, Akt-linked K63-Ub residues coherently were reduced, when compared with those in FKBP51-WT cells (Fig. 3c). In FKBP51 KO cells, ectopic FKBP51 restored K63-ubiquitination of Akt, but it was ineffective when mutated K63R-Ub was added to the system (Fig. 3d). Collectively, these results support the pivotal role of the FKBP51-TPR domain in Akt ubiquitination. To further reinforce the promoting role of FKBP51 on Akt ubiquitination, we performed an in vitro ubiquitination assay (Fig. 3e). After transfection with HA-AKT and HA-TRAF6, HA-IP was obtained from melanoma cells over-expressing or not FKBP51 and was assayed in vitro with the TRAF6-specific Ubc13/Uev1a ubiquitin-conjugating (E2) enzyme. As shown in Fig. 3e, Akt ubiquitination observed in the absence of FKBP51 (lane 3) was remarkably increased in cells overexpressing FKBP51 (lane 1). Relative quantification of bands from Fig. 3 blots is shown (Supplementary Information, Fig. S6).

### PHLPP improves Akt ubiquitination

**Fig. 4:**
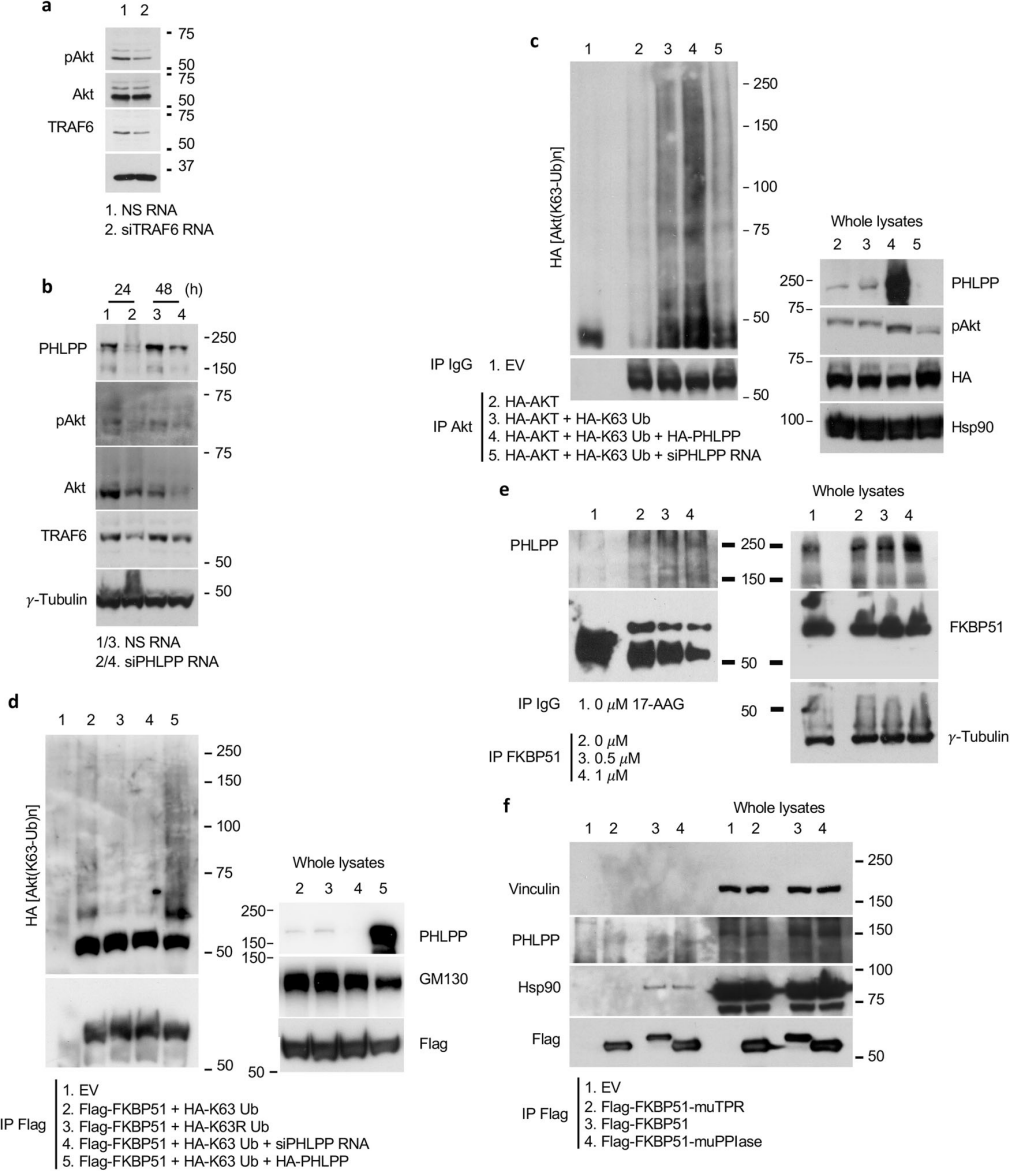
PHLPP improves K63-ubiquitination of Akt. **a** IB assay of A375 cells transfected with siTRAF6 RNA and NS RNA as negative control. Cells were collected after 24 h from transfection. IB shows that silencing of TRAF6 decreased pAkt levels. **b** IB assay of A375 cells transfected with siPHLPP RNA and NS RNA as negative control. Cells were collected after 24 and 48 h from transfection. IB shows that silencing of PHLPP decreased TRAF6 levels. **c** IB analysis of A375 cells transfected with HA-Akt, HA-K63-Ub, HA-PHLPP and siPHLPP RNAs and immunoprecipitated with Akt. IgG served as control for non-specific binding. IB showed that PHLPP increased Akt K63-Ub binding, whereas silencing of the phosphatases decreased it. IB of whole lysates is also shown. **d** IP assay of A375 cells transfected with Flag-FKBP51, HA-PHLPP, HA-K63-Ub and siPHLPP RNAs. Cells were immunoprecipitated with a Flag antibody and IgG served as control for non-specific binding. Immunoprecipitated protein was then assayed by IB with anti-K63-Ub antibody. PHLPP increased K63-Ub binding to FKBP51. IB of whole lysates is also shown. **e** IP assay of A375 melanoma cells treated with 0, 0.5 and 1 µM of the HSP90 inhibitor 17-AAG for 16 h. Endogenous FKBP51 was immunoprecipitated with anti-FKBP51 antibody, while IgG served as control for a not-specific binding. IB showed that inhibition of HSP90 did not affect the binding of FKBP51 to PHLPP. Immunoblot of whole lysates is also shown. **f** IP assay of A375 melanoma cells transfected with Flag-FKBP51, FKBP51-mutPPIase or Flag-FKBP51-mutTPR. Flag-FKBP51 was immunoprecipitated with anti-Flag antibody, while IgG served as control for non-specific binding. Immunoprecipitated proteins were then assayed by IB, and anti-PHLPP antibody revealed that mutated TPR did not affect the binding of FKBP51 to PHLPP. IB of whole lysates is also shown.

The results described so far reveal the paradox of a scaffold protein (FKBP5) that increases Akt activation despite carrying the phosphatase PHLPP. Because TRAF6 stability is regulated by its phosphorylation at threonine 266 (T266) [47, 48], and PHLPP can remove phosphates at threonine residues [49], we hypothesized a role for PHLPP in increasing TRAF6 stability thus supporting Akt ubiquitination. In support of our hypothesis, TRAF6 depletion resulted in impairment of pAkt levels (Fig. 4a). We measured the level of TRAF6 in A375 cells silenced or not for PHLPP (Fig. 4b). We found that PHLPP depletion reduced levels of TRAF6 (Fig. 4b). Then, we investigated the effect of PHLPP on Akt K63-ubiquitination. To this end, we immunoprecipitated Akt from A375 cells overexpressing or silenced for PHLPP (Fig. 4c), and we assayed K63-Ub binding in IB. We observed an increased ubiquitination activity when PHLPP levels were increased, while the silencing of the phosphatase hampered K63 ubiquitination (Fig. 4c). In whole lysates, pAkt levels appeared to be modulated in accordance with Akt ubiquitination. An increased binding of FKBP51 to K63-Ub residues after PHLPP overexpression (Fig. 4d) was consistent with an Akt ubiquitination promoting activity by PHLPP. Given the promoting role of PHLPP in Akt ubiquitination, as Hsp90 appeared to be an essential factor in Akt ubiquitination, we looked at whether PHLPP could be detached from the complex in the absence of Hsp90. To this purpose, we treated the cells with the Hsp90 inhibitor 17-AAG. Immunoprecipitation (IP) of endogenous FKBP51 in presence of 0.5 and 1 μM 17-AAG showed that the inhibitor did not affect PHLPP binding (Fig. 4e). Similarly, mutTPR showed impaired binding to Hsp90 but not to PHLPP (Fig. 4f). Relative quantification of bands from Fig. 3 blots is shown (Supplementary Information, Fig. S7). The inability to promote Akt polyubiquitination process in the absence of Hsp90 suggested that PHLPP is a necessary but not sufficient factor in FKBP51-mediated Akt activation. Taken together, these results support the conclusion that PHLPP participates in Akt polyubiquitination as a factor that promotes the construction of polyubiquitin chain by guaranteeing the stability of the trigger factor, which is the E3-Ub ligase.

### PHLPP drives melanoma cells towards an increased oncogenic behavior

**Fig. 5:**
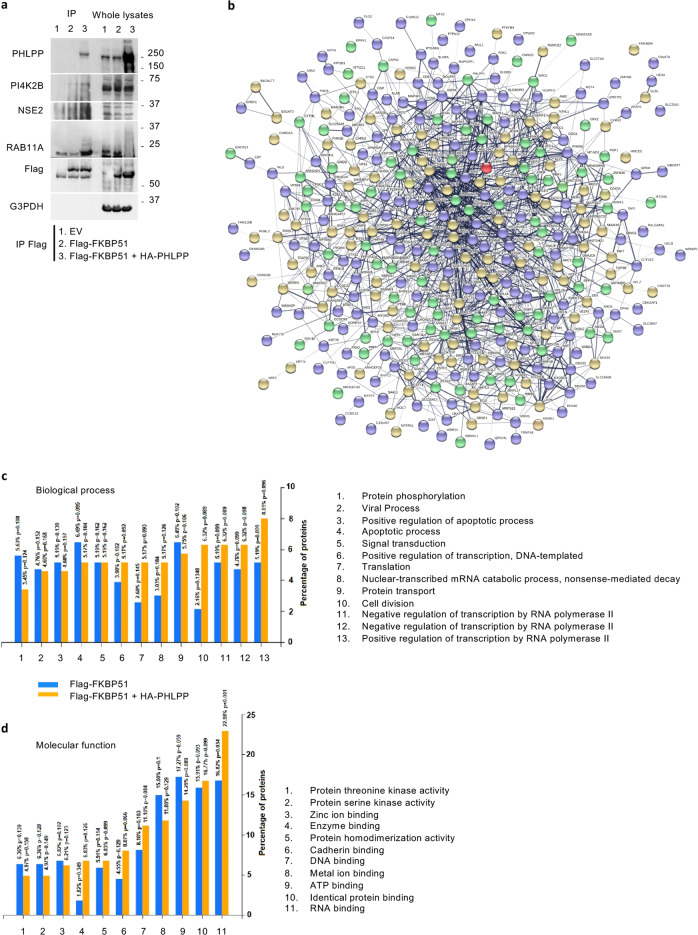
PHLPP sustains the oncogenic function of FKBP51. **a** IP assay of A375 cells transfected with EV, Flag-FKBP51, Flag-FKBP51 + HA-PHLPP. Cells were immunoprecipitated with a Flag antibody and compared to EV as control for non-specific binding. Immunoprecipitated protein was then assayed by IB with the indicated antibodies to validate selected FKBP51 interactors identified by proteomic analysis. PHLPP increased binding of such interactors to FKBP51. IB of whole lysates is also shown. **b** FKBP51-protein interaction network, as deriving from STRING analysis of components identified by proteomic analysis of IB samples from A375 cells in basal and PHLPP-over-representing conditions, respectively (Supplementary Information, Table S1). FKBP51 is highlighted in red; direct/indirect interactors identified in basal, PHLPP-over-representing or both conditions are reported in blue, yellow and green, respectively. **c** Functional annotation of the global network based on Gene Ontology-Biological Process terms. **d** Functional annotation of the global network based on Gene Ontology-Molecular function terms.

Our findings suggested that PHLPP, by assisting FKBP51-mediated Akt ubiquitination, can promote the pro-oncogenic function of the immunophilin. With the aim to investigate the putative molecular scenario(s) by which PHLPP may exert this activity, we performed a proteomic analysis to identify direct/indirect FKBP51 interactors associated with the presence or not of this phosphatase. To this purpose, we analyzed the proteomic profile of Flag-FKBP51 IP samples obtained from A375 cells in a condition or not of over-representation of PHLPP. Overall, 382 and 304 putative FKBP51-interacting proteins were identified in basal and PHLPP-over-representing conditions, respectively (Supplementary Information, Table S1); 121 were common to both experimental setups. The high number of detected FKBP51 interactors in both cases was rationalized according to the chaperone character of this immunophilin, in agreement with previous studies cumulatively describing about 258 non-redundant FKBP51 interactors, as reported in BIOGRID, INTACT, STRING, DIP, STRING and CORUM database. Among interactors here identified, almost two dozen were already described in previous investigations on the FKBP51 interactome [50]. Among novel proteins here ascertained binding to FKBP51, worth mentioning are a number of receptors, kinases, phosphatases, melanoma antigens and components related to transcription, translation and the ubiquitin-related processing machineries, which were often associated with oncogenic hallmarks (Supplementary Information, Table S1). An experimental confirmation of novel FKBP51 interactors here described was obtained by IP experiments, as in the case of PI4K2B, NSE2 and Rab11a (Fig. 5a and Supplementary information, Fig. S8).

All FKBP51-interacting proteins identified in this study were subjected to bioinformatic analysis to identify peculiar molecular networks and/or biological processes related to the presence of basal or over-represented PHLPP levels. Figure 5a describes the network obtained with STRING software; a unique main network was identified containing 364 interaction protein nodes. They are reported with different color depending on their peculiar and common behavior with respect to PHLPP over-representation. No specific network portions were colored differently depending or not on the phosphatase occurrence. Regarding the biological processes that showed significant differences among the two protein interactor datasets (Fig. 5b), FunRich analysis of FKBP51-interacting proteins in basal and PHLPP-over-representing conditions highlighted a certain over-representation in the latter case of components involved in cell division, transcription and translation, along with a decrease of those related to apoptosis. Regarding molecular function, significant differences were detected only for components involved in enzyme, RNA and DNA binding (Fig. 5c).

**Fig. 6:**
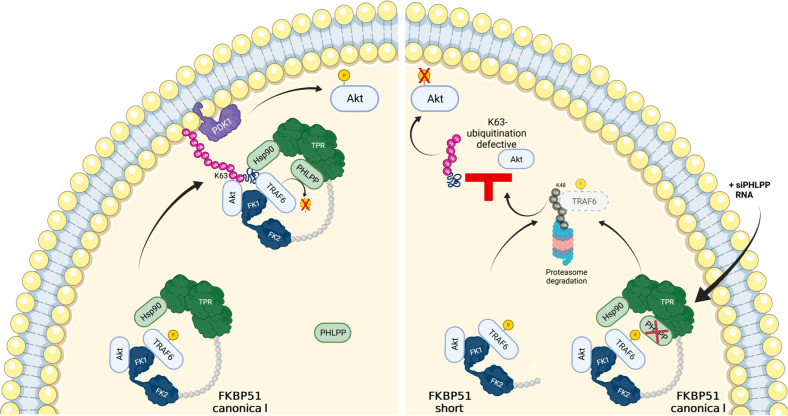
Proposed mechanism for the interaction of FKBP51 with Akt and PHLPP in melanoma cells. Left, FKBP51 binds to PHLPP thus stabilizing TRAF6 and allowing the formation of a K63 polyubiquitin chain and the full activation of Akt. Right, PHLPP is not kept into the complex by FKBP51s, which hampers Akt ubiquitination and phosphorylation. The same occurs when PHLPP is subtracted from the FKBP51 complex.

In conclusion, our results suggest that the FKBP51/Akt/PHLPP machinery promotes Akt phosphorylation, with FKBP51 serving as a scaffold to facilitate kinase K63-polyubiquitination, and PHLPP serving to stabilize the E3 ubiquitin ligase TRAF6, as graphically summarized in Fig. 6. Although preliminary proteomic evidence suggest a role of PHLPP as a promoter of FKBP51 pro-oncogenic action, further studies are necessary to describe punctually the molecular determinants/machineries involved in this activity as well as the corresponding mechanisms.

## Discussion

FK506 Binding Proteins (FKBPs), known as intracellular ligands of immunosuppressant agents, are highly conserved across the species and abundantly expressed in all organisms, with defined cellular roles in multiple signaling pathways even in the absence of ligands [51]. In the last two decades, several studies identified some FKBPs as active players in cancer, raising interest in these proteins as possible tumor targets and biomarkers [51]. In this context, FKBP51 is an immunophilin physiologically expressed in lymphocytes and several other tissues, where it is an essential factor for cell proliferation and survival [52, 53]. Its relevant role in sustaining cancer cell growth and aggressiveness has been documented in various human malignancies, where it is deregulated and sustains tumor resistance and aggressiveness [18, 46, 51, 53–59]. FKBP51 binds to Hsp90 through its TPR domain and functions as co-chaperone in Hsp90-mediated functions [60]. Hsp90 interacts with and stabilizes Akt [61] and, particularly, it promotes Akt and PDK1 association by physically interacting with both [4, 47]. The overt oncogenic role of FKBP51 contrasts with the highlighted role of this immunophilin in supporting PHLPP in Akt inactivation [9]. The recent identification of the spliced isoform of FKBP51 [17, 39], namely FKBP51s, which lacks the TPR domain and is unable to bind to Hsp90, suggested that it might act as a dominant-negative, being unable to support Akt activation.

In this study, we show that FKBP51 and FKBP51s act differently on Akt activation. Precisely, the canonical isoform promotes Akt phosphorylation and activation of its downstream substrates, namely P70S6k and cyclin D1, whereas FKBP51s is unable to foster Akt activation and can even promote de-phosphorylation of this kinase. The observation that the spliced FKBP51 can bind to Akt, but not to PHLPP, ruled out a role for such a phosphatase in reduced pAkt levels observed upon ectopic FKBP51s expression, in comparison with levels induced by ectopic expression of FKBP51.

We also found that FKBP51 strongly binds to K63-ubiquitin residues and not to K48-ubiquitin. Conversely, we observed that FKBP51s do not interact with K63-ubiquitin, suggesting it loses this capability. Consistent with such finding, FKBP51 overexpression enhanced the K63-ubiquitination of Akt, supporting a role for this scaffold protein in the Akt ubiquitination process. Like FKBP51s, the FKBP51-TPRmut (carrying the point mutation K352A/R356A hampering the binding to Hsp90) did not promote Akt K63-ubiquitination and activation. Instead, FKBP51-PPIasemut maintained the capability of upregulating Akt activation. These findings highlighted a central role of the TPR scaffold domain of FKBP51 in Akt activation, while excluding a role for the isomerase function of FKBP51. The pharmacological inhibition of Hsp90 strongly impaired K63-ubiquitination of Akt, suggesting that this chaperone is essential for FKBP51-mediated Akt activation. In this context, Sato and coworkers previously demonstrated the essential role of Akt-Hsp90 interaction in safeguarding the enzymatic function of the kinase [47]. These authors also pointed out to PP2A as a major effector for controlling the amount of phospho-Akt [47]. The involvement of FKBP51 in the K63-ubiquitination of Akt was even more stringent using FKBP51 KO clones in which Akt ubiquitination was impaired and restored following the FKBP51 rescue. Interestingly, this recovery was unsuccessful when we have supplied the system with mutated K63-ubiquitin. By modulating the level of PHLPP, we were able to clarify the role of this phosphatase, which was also found linked to the FKBP51/Akt/K63-ubiquitin/Hsp90 complex. Surprisingly, PHLPP upregulation even increased pAkt levels. Such a condition improved the efficiency of K63-ubiquitination of Akt. Conversely, the specific silencing of PHLPP produced an opposite effect. The finding that the silencing of this phosphatase decreased TRAF6 levels suggested that PHLPP can promote Akt K63-ubiquitin linkage through stabilization of such E3 Ub-ligase, which is a known FKBP51 interactor [20]. Phosphorylation at Thr266, indeed, promotes proteasome-mediated degradation of TRAF6 [62]. Interestingly, FKBP51 binding to TRAF-C domain of TRAF6 [20] does not prevent the binding of other proteins that contain TRAF family consensus motifs, thus allowing to form complexes with other TRAFs such as TRAF2 [14, 63], in line with a scaffolding function for FKBP51. Our results suggested a novel and unexplored role for PHLPP in promoting Akt K63-ubiquitination by sustaining TRAF6-E3 ligase activity in a melanoma cell context.

Above-mentioned hypothesis was supported by a proteomic analysis that compared the interactome of FKBP51 in condition or not of PHLPP overexpression. A bioinformatic analysis of obtained results clearly delineated a scenario of increased cell division, which was accompanied by an enhanced activation of the transcription/translation machinery in presence of high PHLPP levels, along with a decreased apoptosis (Fig. 5c). Even if PHLPP is generally considered as a tumor-suppressor [64], increasing evidence also support a pro-tumoral role for this phosphatase [65–67]. By suggesting a pivotal role of PHLPP in the FKBP51/TRAF6/Akt complex, our results are only apparently in contrast with those reporting that FKBP51 overexpression in pancreatic cancer lowers pAkt levels [9, 68–71]. Indeed, this can happen in systems in which PHLPP is barely expressed (Supplementary information, Fig. S9). In line with this hypothesis, an analysis of PHLPP levels in different cancer tissues from the Human Protein Atlas data bank (https://www.proteinatlas.org/ENSG00000081913-PHLPP1/pathology) showed that PHLPP is highly expressed in tumors as melanoma and glioma, while its levels are negligible in some tumors, including pancreatic cancer (Supplementary information, Fig. S9a), as also suggested by an immunoblot assay (Supplementary information, Fig. S9b). The same immunoblot (Fig. S9b) shows that levels of TRAF6 are comparable in the pancreatic SU86 and melanoma A375 cell lines. This result is consistent with those retrieved from the Human Protein Atlas data bank (https://www.proteinatlas.org/ENSG00000175104-TRAF6/pathology) (Supplementary information, Fig. S9c). Regulation of FKBP51 scaffold function by post-translational modifications could be an additional explanation of the diversity in the effects of FKBP51 on Akt activation in relation to the cancer-cell context. Park et al. found a reduced interaction of phosphorylated-FKBP51 with Akt [71]. Zgajnar et al. identified more than one phosphorylation site for FKBP51, therefore it cannot be excluded that phosphorylation/dephosphorylation of a not yet identified residue can modulate FKBP51 capability of activating Akt [72]. A post-translational modification was also reported by Yu at al. [19] that found that the FKBP51 acetylation by P300/CBP increased pSer^473^Akt levels.

In conclusion, the present work provides novel insights into the role of the previously identified FKBP51/Akt/PHLPP complex. We demonstrated this protein-complex operates in K63-ubiquitination of Akt, thus highlighting a novel oncogenic function for PHLPP. A low tumor expression level of PHLPP could not support Akt activation in condition of FKBP51-overexpression. Heterogeneity in PHLPP expression or post-translational modifications of FKBP51 in different tumor contexts can explain the diverging results reported in the scientific literature regarding the role of FKBP51 in the regulation of Akt phosphorylation.

## Supplementary information

Supplementary information

Original Data File

Table S1

aj-checklist

## Data Availability

Source data underlying figures are presented in Supplementary information. Data underlying the inventory reported in Supplementary Information (Table S1) are available via ProteomeXchange with identifier PXD033828. All other data from this study are available from the corresponding author upon reasonable request.
