# Supplementary material for: FKBP51 plays an essential role in Akt ubiquitination that requires Hsp90 and PHLPP
Source: Cell Death Dis. 2023 Feb 13;14(2):116. doi: 10.1038/s41419-023-05629-y (PMC9925821; doi:10.1038/s41419-023-05629-y)
Supplement: Supplementary file 1 — Supplementary information [file 41419_2023_5629_MOESM1_ESM.pdf]

Supplementary Information

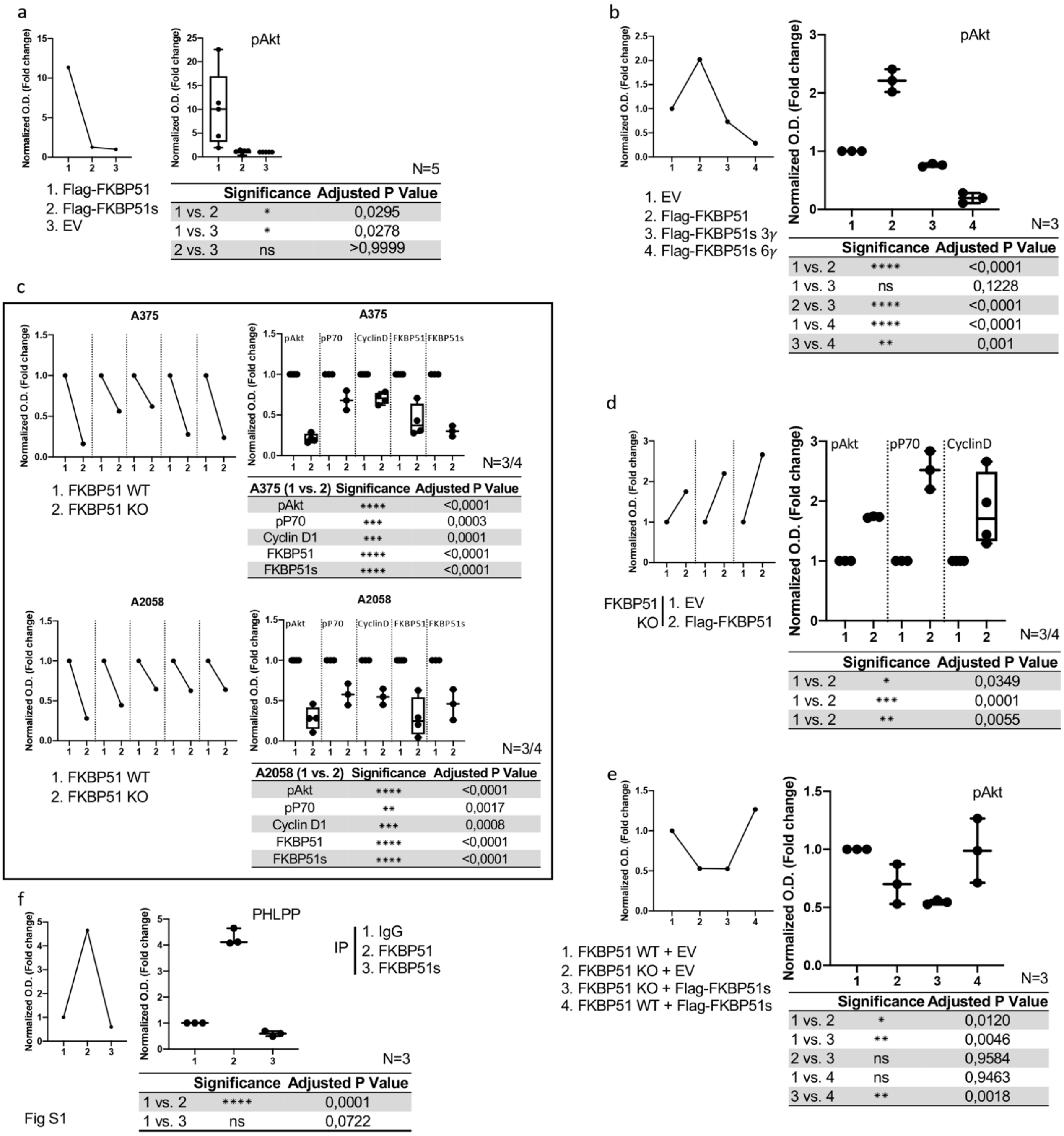

Figure S1. (a-e) Densitometric analysis of IB from Figure 1 (a-e). Bands were quantitated by densitometry, using ImageJ 1.42q for Macintosh. Integrated OD were normalized to a relative housekeeping gene and expressed as fold increase, arbitrarily using the control of each experiment as reference sample (expression =1). The data are shown as graph of single values relative to the main IB (left) and as mean ± SEM (right); unless otherwise indicated, presented data are representative results of at least three independent repeats. Densitometry of panel f includes the three biological replicates of Figure 1f and Figure S2d. Statistical analyses were performed using GraphPad Prism 7, and the statistics were analyzed by two-tailed Student's t test or one-way or two-way ANOVA. Differences were considered to be significant at $P \leq 0.05$ and are indicated by *; those at $P \leq 0.01$ are indicated by **; those at $P \leq 0.001$ are indicated by *** and those at $P \leq 0.0001$ are indicated by ****.

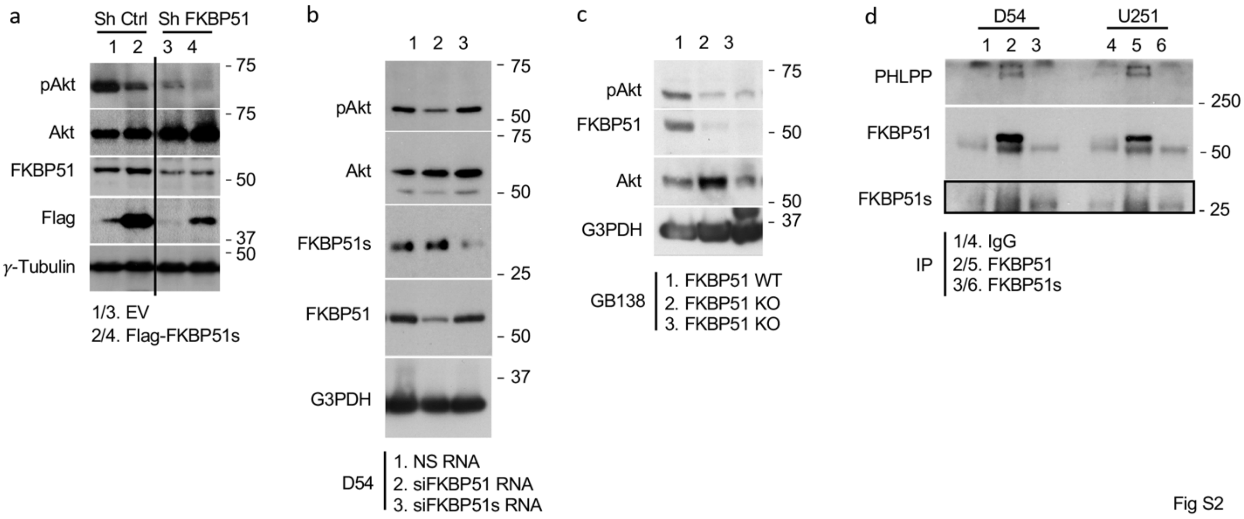

Figure S2. (a) IB of A375 cells stably knocked down with a FKBP51 shRNA (Sh FKBP51) or with a control shRNA (Sh Ctrl). Cells were transfected with Flag-FKBP51s or EV as control. PAkt levels were impaired in Sh FKBP51 cells, compared to Sh Ctrl cells; FKBP51s overexpression further decreased Akt activation levels. γ-Tubulin was used as loading control. Ectopic FKBP51s impaired pAkt levels. (b) IB assay of D54 glioblastoma cells transfected with specific siRNAs for FKBP51 and FKBP51s, or a NS RNA as control. Silencing of the canonical isoform affected pAkt levels, whereas silencing of the short isoform did not. G3PDH was used as loading control. (c) GB138 glioblastoma cells stably knocked out for FKBP51 with the CRISPR/Cas9 technology. IB assay showed impaired levels of pAkt in KO cells compared to control (WT) cells. G3PDH was used as loading control. (d) IP of endogenous FKBP51 isoforms in D54 and U251 glioblastoma cells. IgG served as control for a non-specific binding. Immunoprecipitated proteins were then assayed by IB, and anti-PHLPP antibody revealed that PHLPP was bound only to the canonical FKBP51.

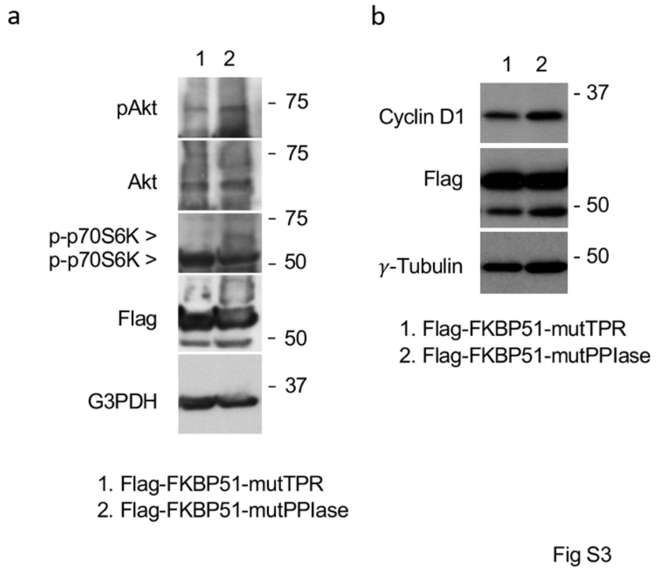

Figure S3. IB of Akt/P70S6k activation (a) and cyclin D1 expression (b) in mutTPR and mutPPIase. A375 cells were transfected with Flag-FKBP51-mutTPR or with Flag-FKBP51-mutPPIase. IB analysis showed that TPR activity inhibition impairs pAkt and p-P70S6k levels. G3PDH and γ-tubulin were used as loading control.

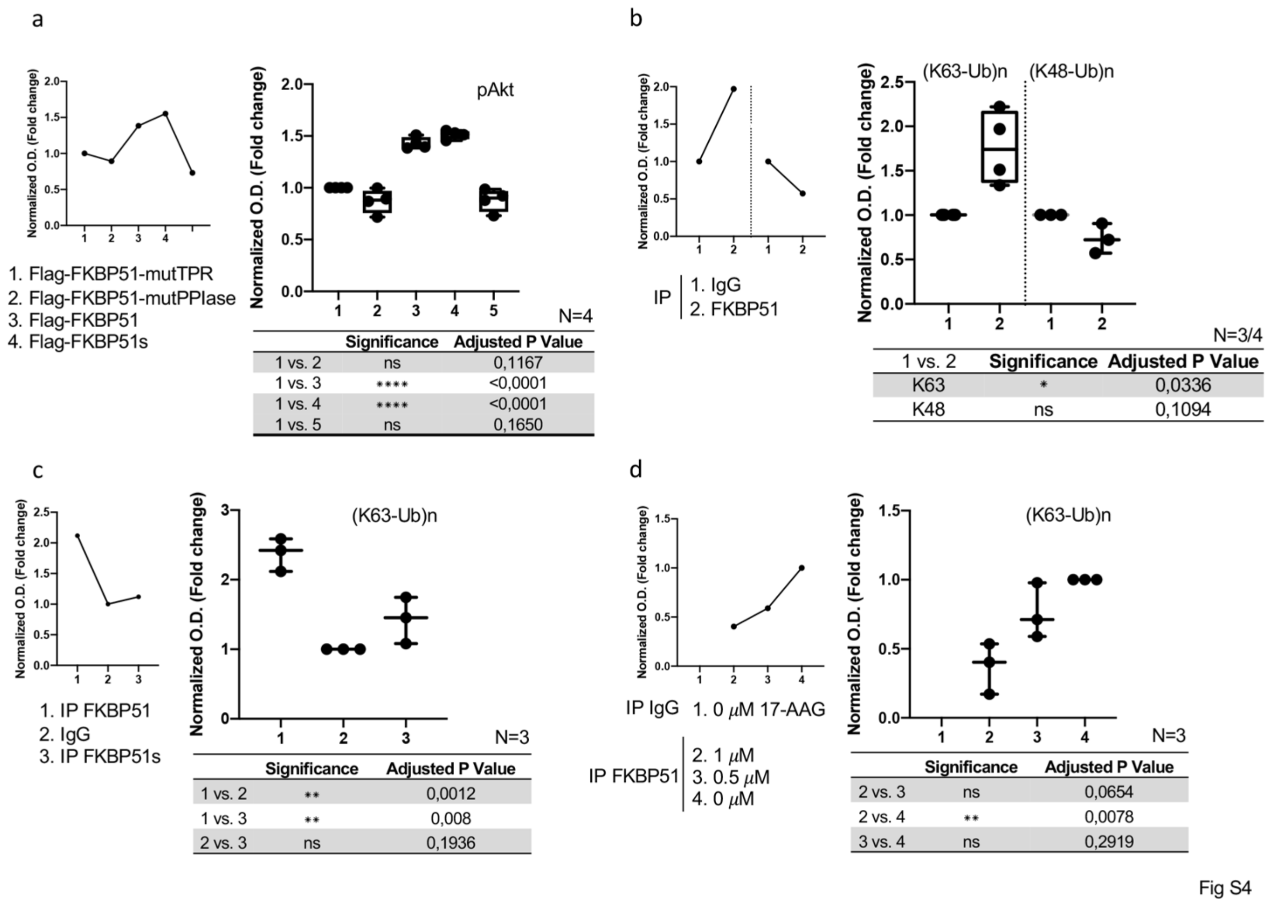

Figure S4. (a-d) Densitometric analysis of IB from Figure 2 (a-d). Densitometric analysis of IB from Figure 2a. Bands were quantitated by densitometry, using ImageJ 1.42q for Macintosh. Integrated OD were normalized to γ-tubulin and expressed as fold increase, arbitrarily using Flag-FKBP51 as reference sample (expression =1). The data are shown as graph of single values relative to the main IB (left) and as mean ± SEM (right); unless otherwise indicated, presented data are representative results of at least two or three independent repeats. Statistical analyses were performed using GraphPad Prism 7, and the statistics were analyzed by two-tailed Student's t test or one-way or two-way ANOVA. Differences were considered to be significant at $P \leq 0.05$ and are indicated by *; those at $P \leq 0.01$ are indicated by **; those at $P \leq 0.001$ are indicated by *** and those at $P \leq 0.0001$ are indicated by ****.

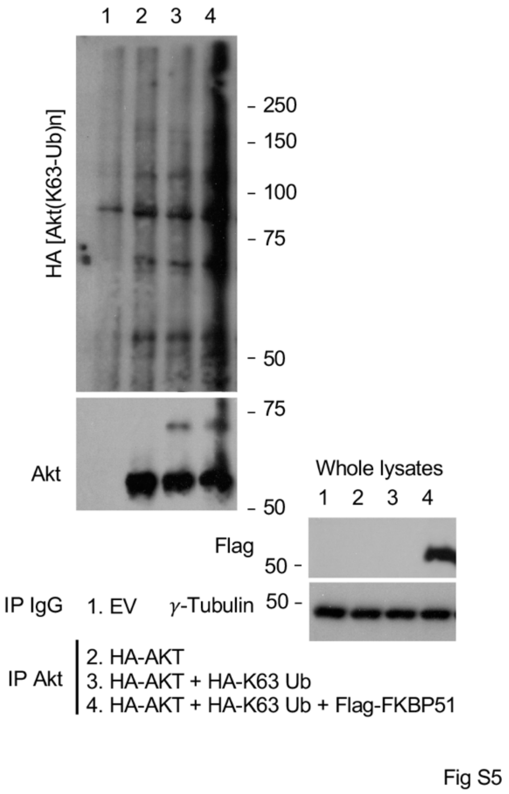

Figure S5. IP assay of A2058 melanoma cells transfected with HA-Akt, HA-K63-Ub and Flag-FKBP51. Immunoprecipitated protein was then assayed by IB. Anti-HA antibody revealed an increased K63-linked ubiquitination of Akt upon FKBP51 overexpression. IB of whole lysates is also shown; γ-tubulin was used as loading control.

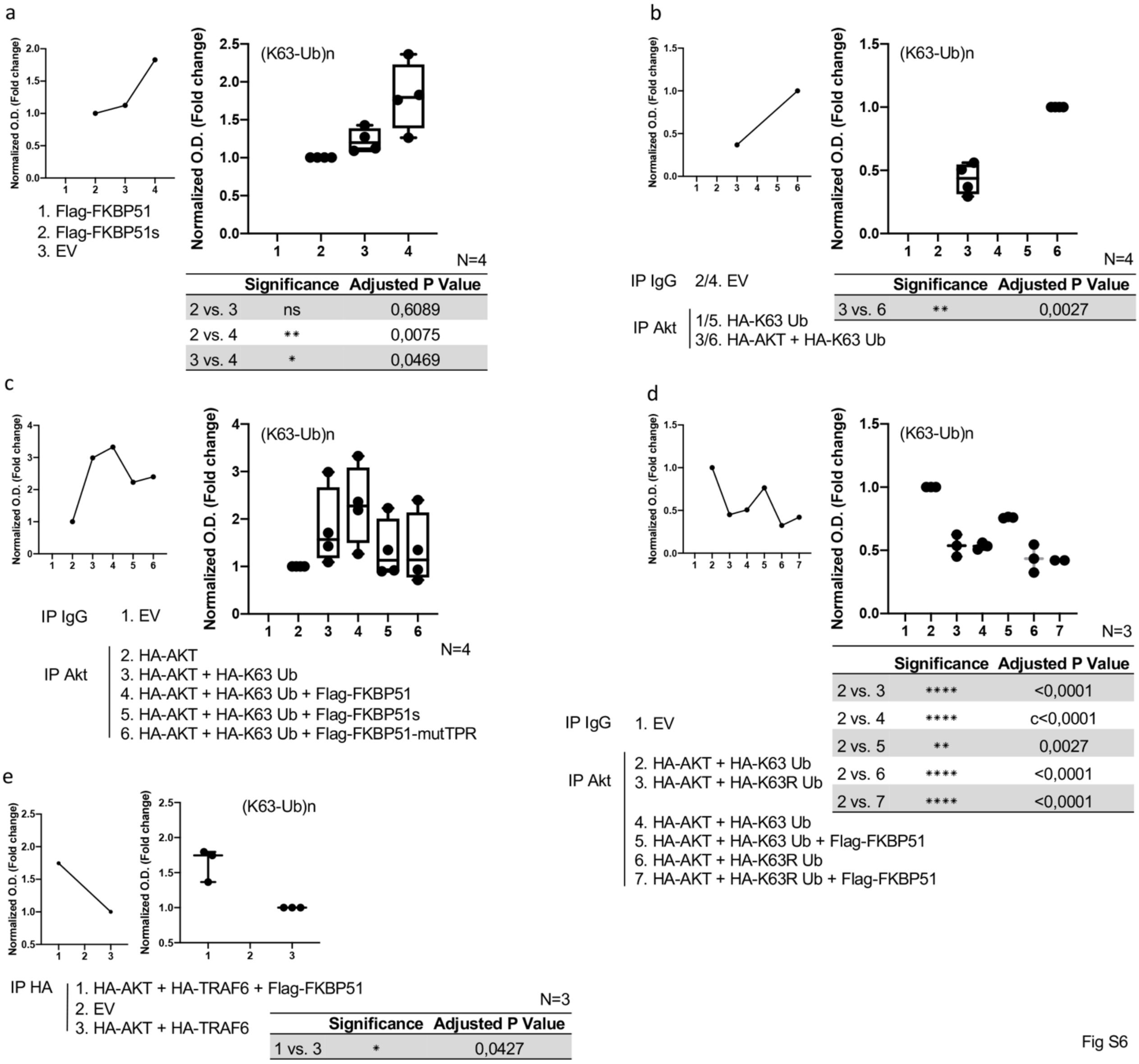

a

	Significance	Adjusted P Value
2 vs. 3	ns	0,6089
2 vs. 4	**	0,0075
3 vs. 4	*	0,0469

b

	Significance	Adjusted P Value
3 vs. 6	**	0,0027

d

	Significance	Adjusted P Value
2 vs. 3	****	<0,0001
2 vs. 4	****	c<0,0001
2 vs. 5	**	0,0027
2 vs. 6	****	<0,0001
2 vs. 7	****	<0,0001

e

	Significance	Adjusted P Value
1 vs. 3	*	0,0427

Figure S6. (a-e) Densitometric analysis of IB from Figure 3 (a-e). Bands were quantitated by densitometry, using ImageJ 1.42q for Macintosh. Integrated OD were normalized to a relative housekeeping gene and expressed as fold increase, arbitrarily using the control of each experiment as reference sample (expression =1). The data are shown as graph of single values relative to the main IB (left) and as mean ± SEM (right); unless otherwise indicated, presented data are representative results of at least three independent repeats. Statistical analyses were performed using GraphPad Prism 7, and the statistics were analyzed by two-tailed Student’s t test or one-way or two-way ANOVA. Differences were considered to be significant at P ≤ 0.05 and are indicated by *; those at P ≤ 0.01 are indicated by **; those at P ≤ 0.001 are indicated by *** and those at P ≤ 0.0001 are indicated by ****.

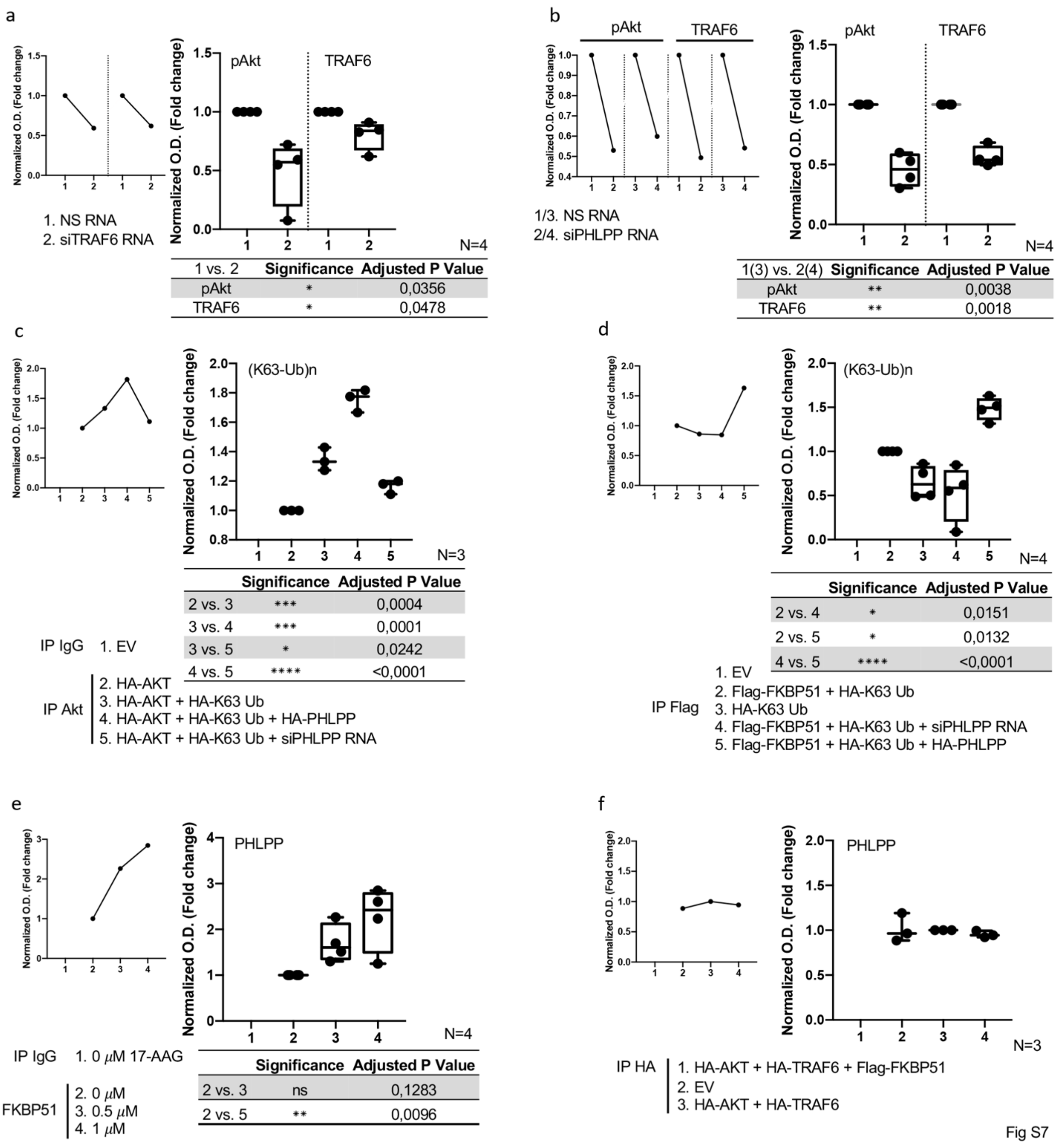

Figure S7. (a-f) Densitometric analysis of IB from Figure 4 (a-f). Bands were quantitated by densitometry, using ImageJ 1.42q for Macintosh. Integrated OD were normalized to a relative housekeeping gene and expressed as fold increase, arbitrarily using the control of each experiment as reference sample (expression =1). The data are shown as graph of single values relative to the main IB (left) and as mean ± SEM (right); unless otherwise indicated, presented data are representative results of at least three independent repeats. Statistical analyses were performed using GraphPad Prism 7, and the statistics were analyzed by two-tailed Student's t test or one-way or two-way ANOVA. Differences were considered to be significant at $P \leq 0.05$ and are indicated by *; those at $P \leq 0.01$ are indicated by **; those at $P \leq 0.001$ are indicated by *** and those at $P \leq 0.0001$ are indicated by ****.

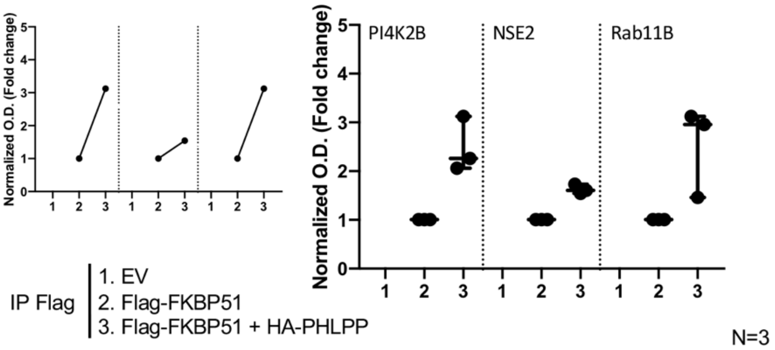

2 vs. 3	Significance	Adjusted P Value
PI4K2B	*	0,0452
NSE2	**	0,0077
Rab11B	ns	0,1035

Fig S8

Figure S8. Densitometric analysis of IB from Figure 5a. Bands were quantitated by densitometry, using ImageJ 1.42q for Macintosh. Integrated OD were normalized to a relative housekeeping gene and expressed as fold increase, arbitrarily using Flag-FKBP51 as reference sample (expression =1). The data are shown as graph of single values relative to the main IB (left) and as mean ± SEM (right); presented data are representative results of three independent repeats. Statistical analyses were performed using GraphPad Prism 7, and the statistics were analyzed by one-way ANOVA. Differences were considered to be significant at $P \leq 0.05$ and are indicated by *; those at $P \leq 0.01$ are indicated by **.

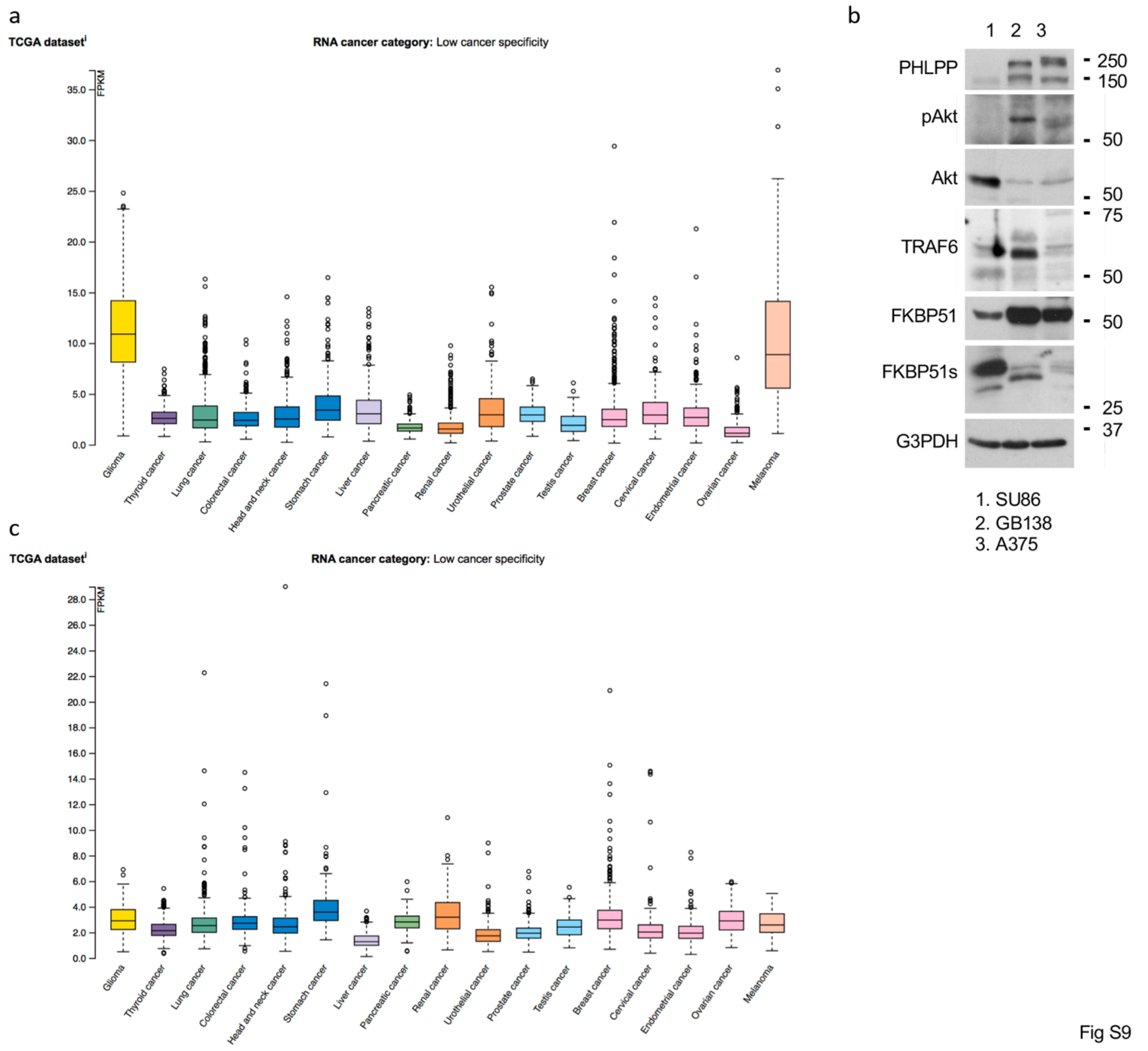

Figure S9. (a) Boxplot representation of PHLPP1 gene expression in different tumors, as downloaded from https://www.proteinatlas.org/ENSG00000081913-PHLPP1/pathology. RNA-seq data were from The Cancer Genome Atlas. (b) IB of PHLPP, pAkt, TRAF6 and FKBP51 isoforms in three cancer cell lines (SU87, GB138 and A375). G3PDH was used as loading control. (c) Boxplot representation of TRAF6 gene expression in different tumors, as downloaded from https://www.proteinatlas.org/ENSG00000175104-TRAF6/pathology. RNA-seq data were from The Cancer Genome Atlas.

Table S1. Identification details of the proteins identified in this study. Reported are the information on protein false discovery rate (FDR) confidence, protein accession, description, gene name, exp. q-value, sequence coverage (%), number of identified peptides, peptide spectrum matches (PSMs), number of identified unique peptides, number of amino acids, molecular mass, pI, Mascot identification score values, protein groups and modification(s).
