# Supplementary figures and images for: FKBP51 plays an essential role in Akt ubiquitination that requires Hsp90 and PHLPP
Source: Cell Death Dis. 2023 Feb 13;14(2):116. doi: 10.1038/s41419-023-05629-y (PMC9925821; doi:10.1038/s41419-023-05629-y)

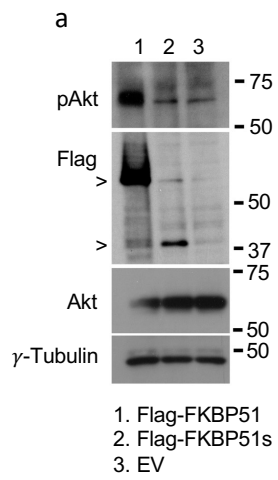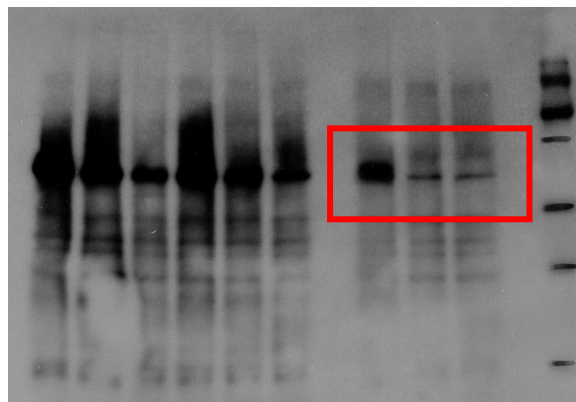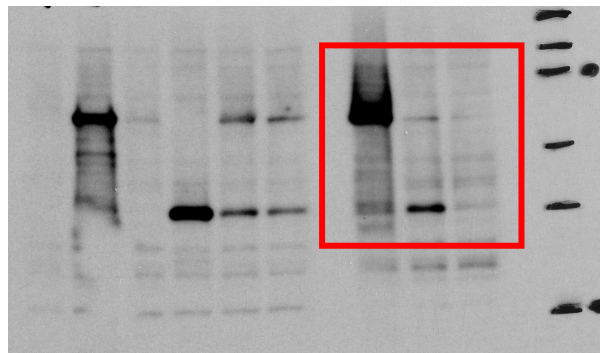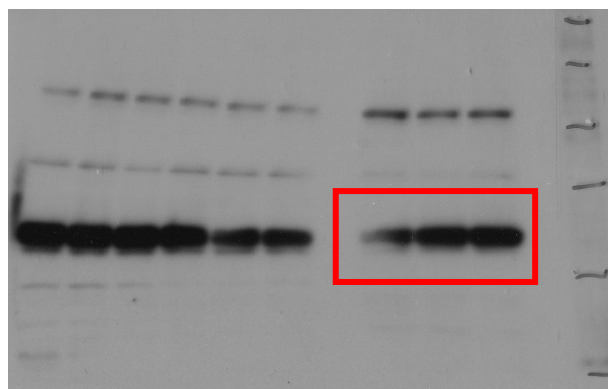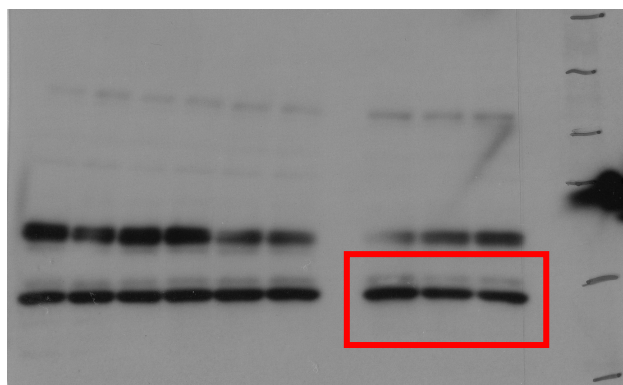

Fig 1

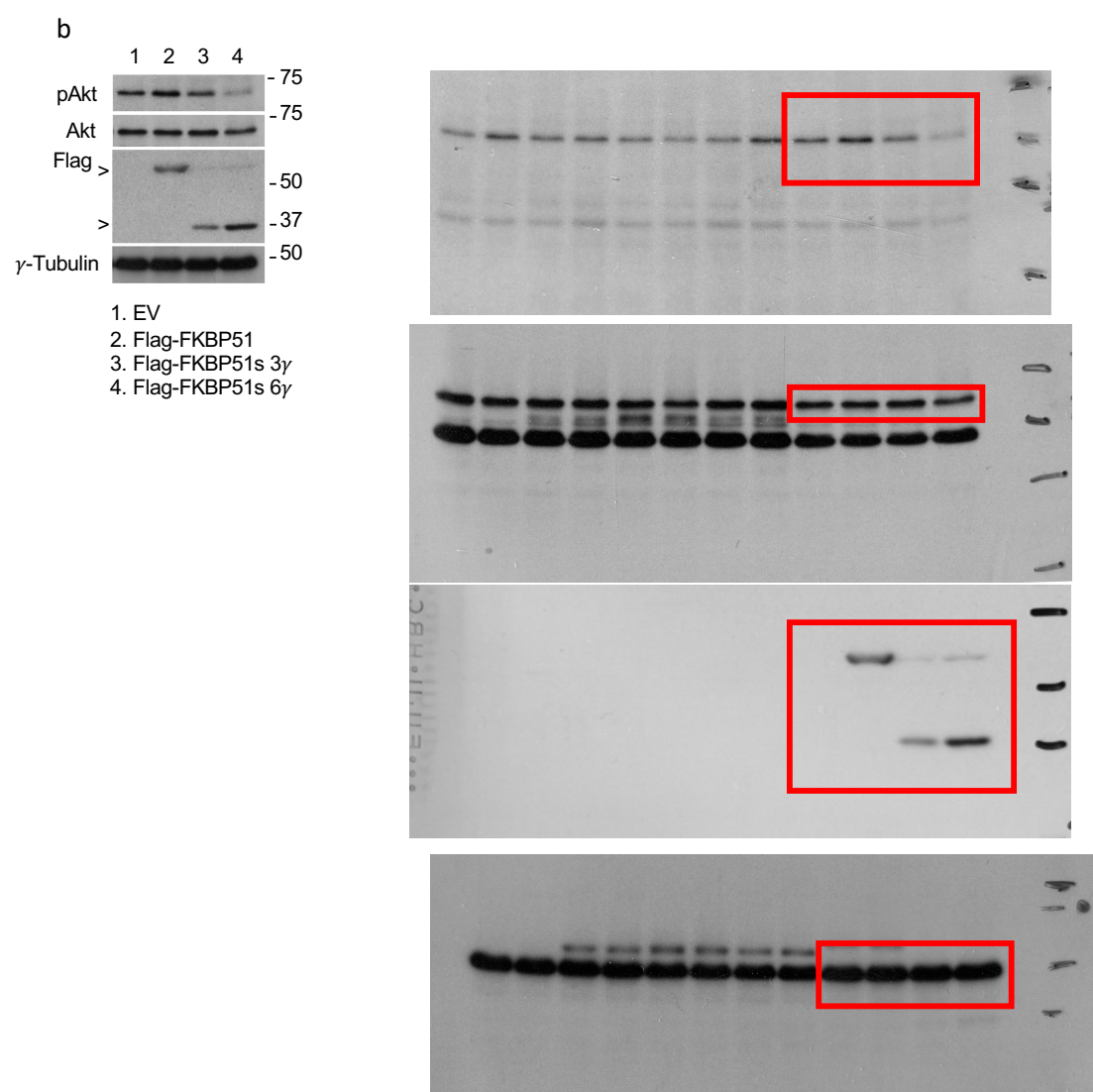

Fig 1

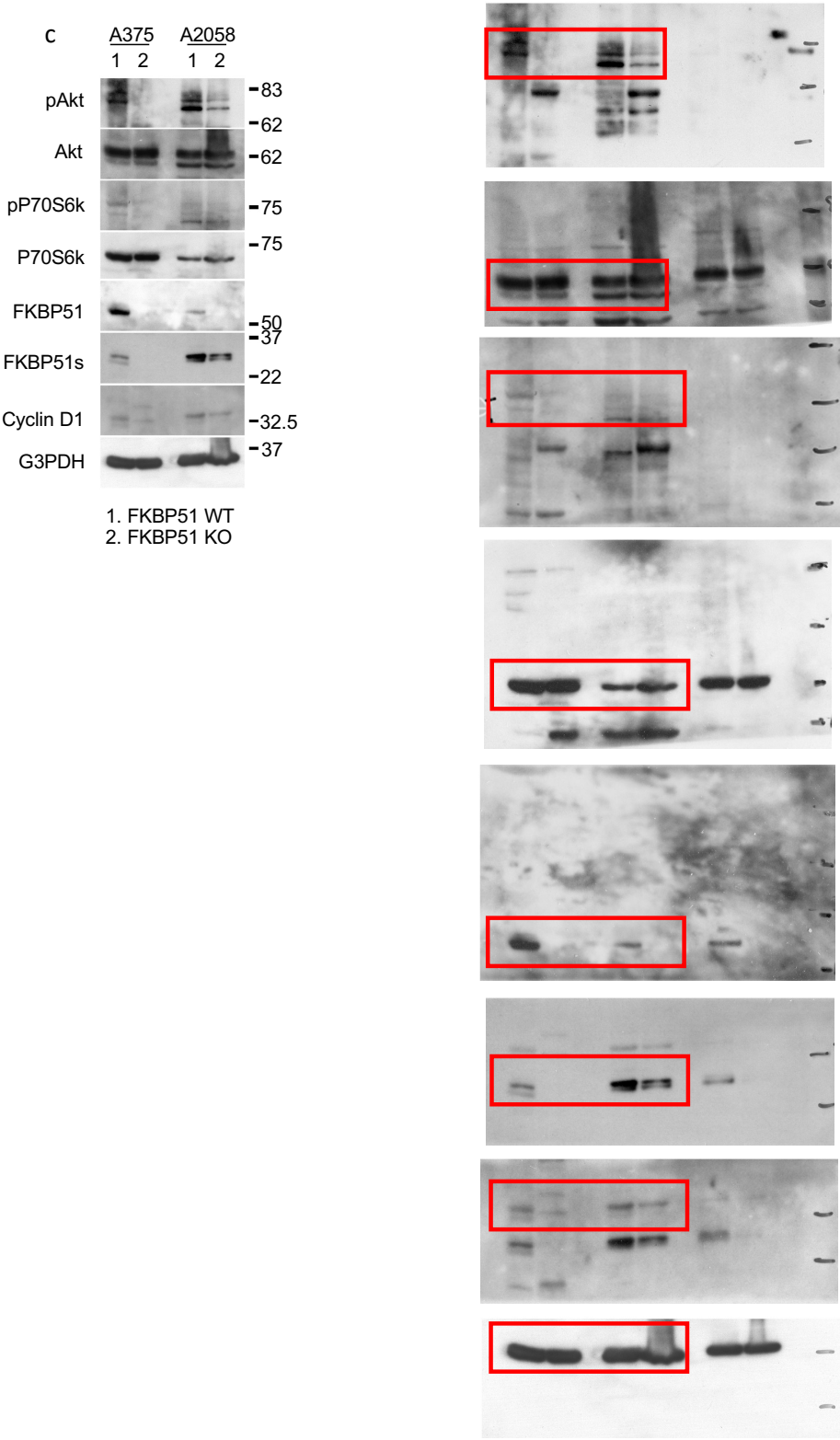

Fig 1

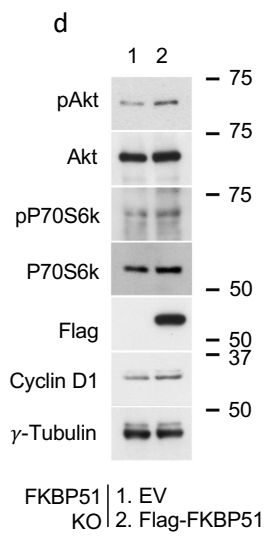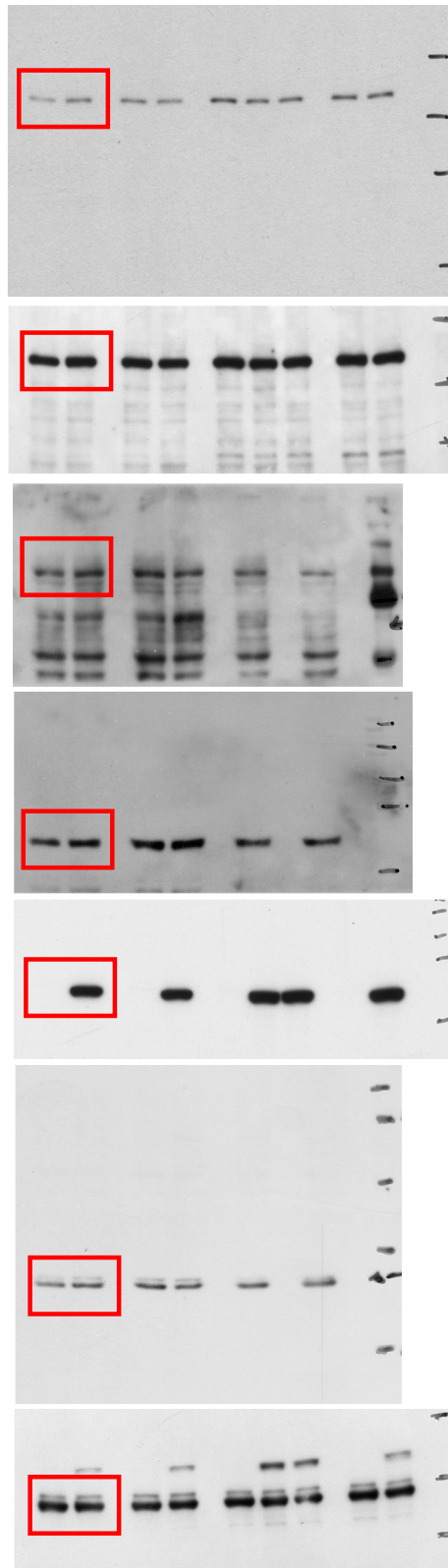

Fig 1

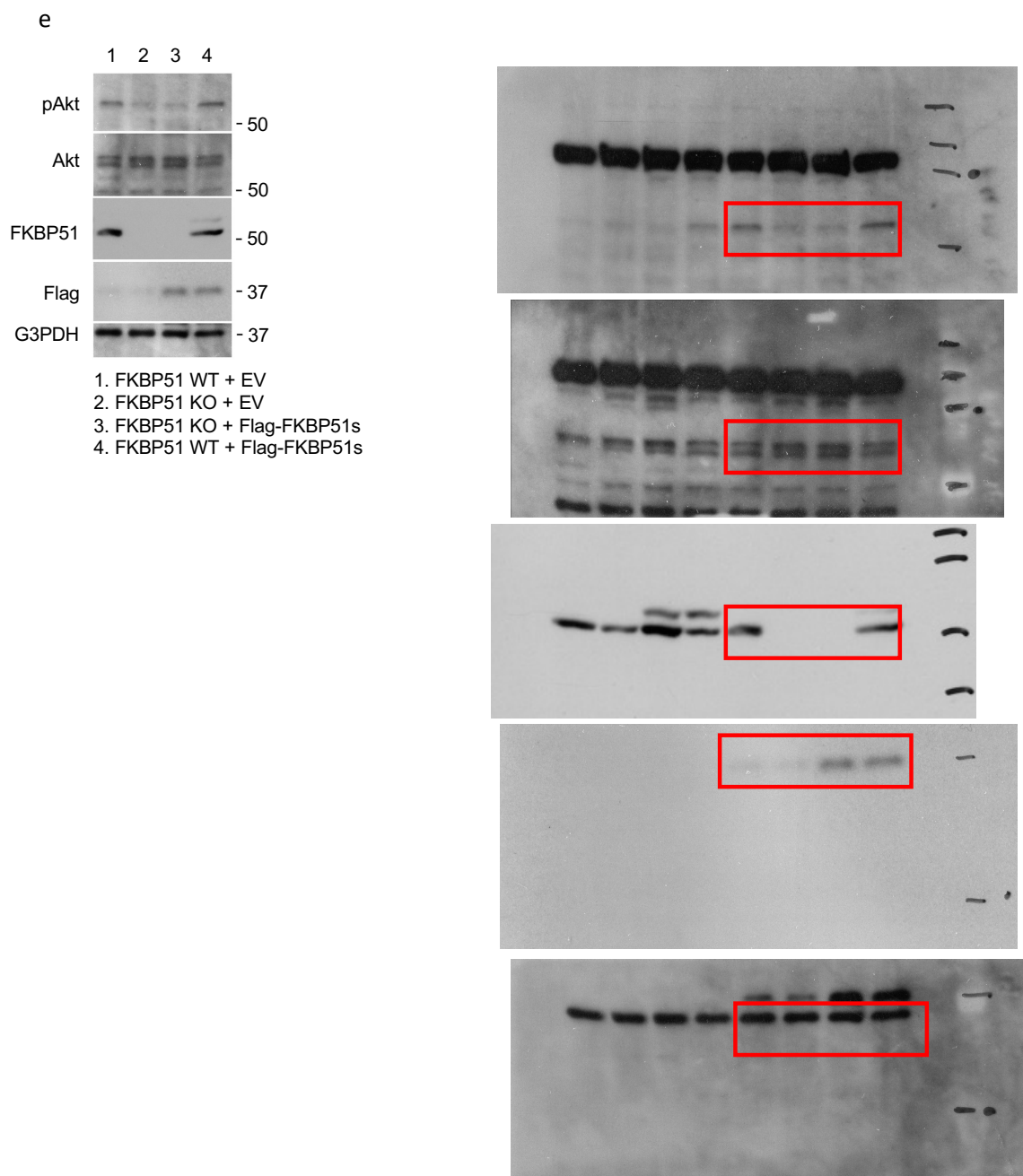

Fig 1

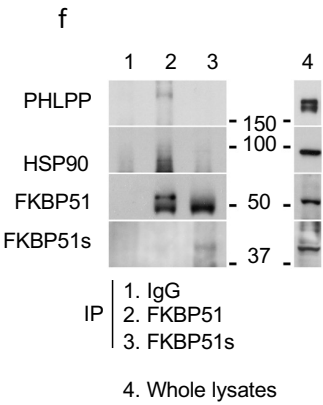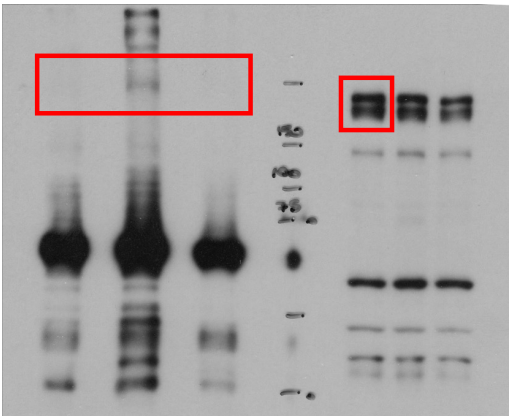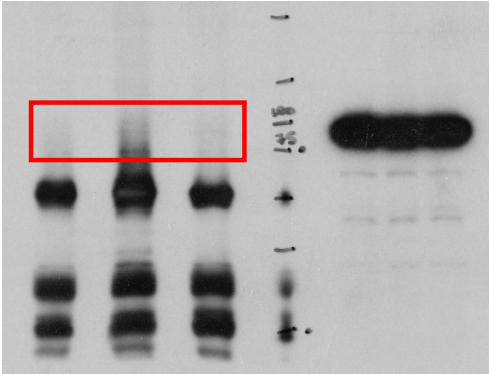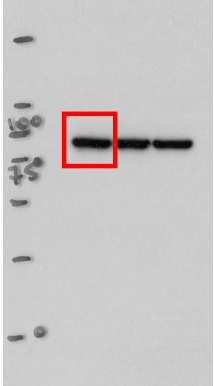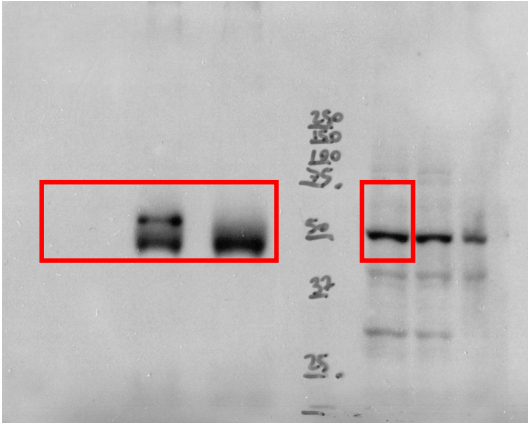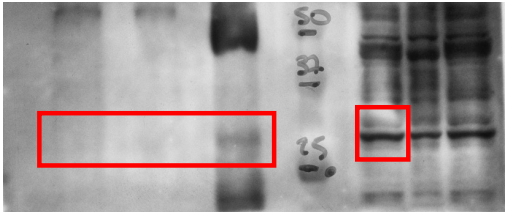

Fig 1

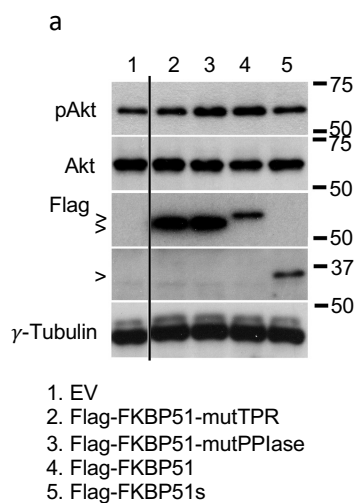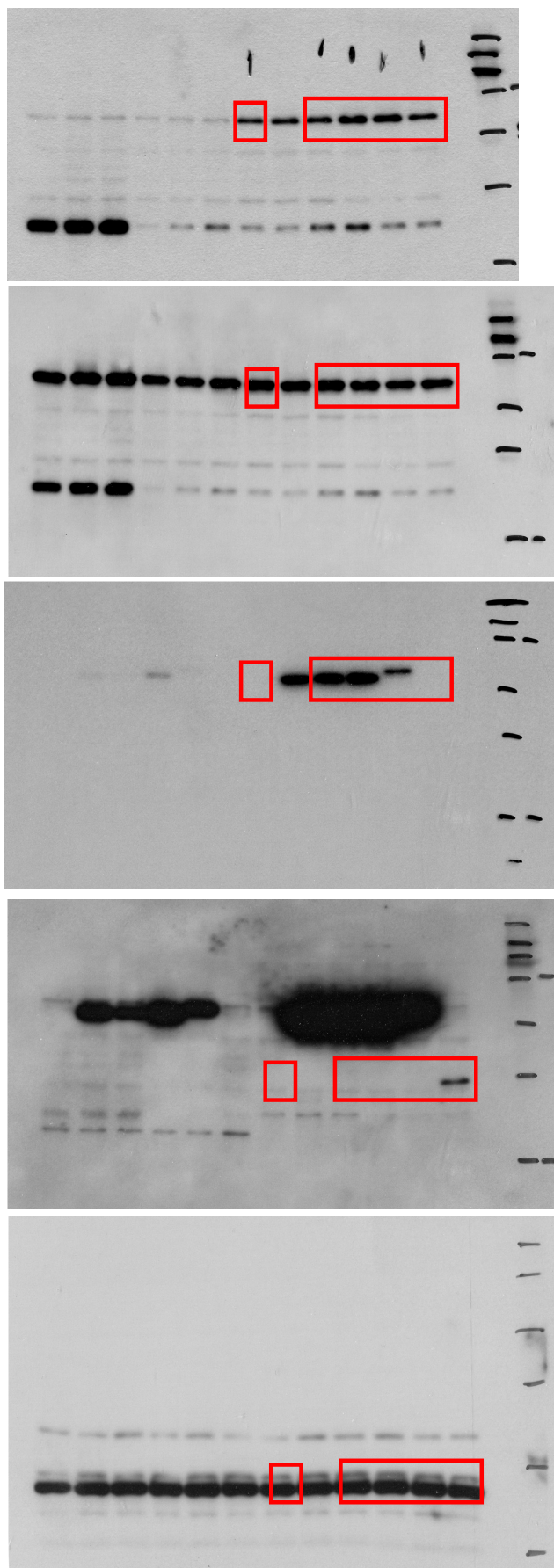

Fig 2

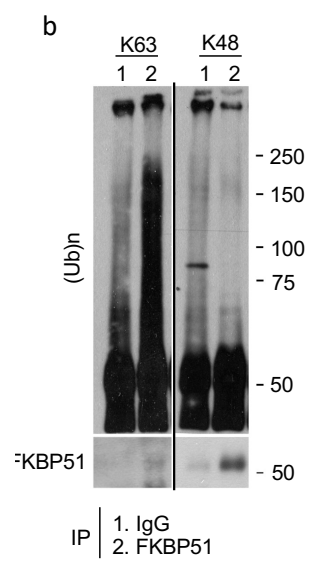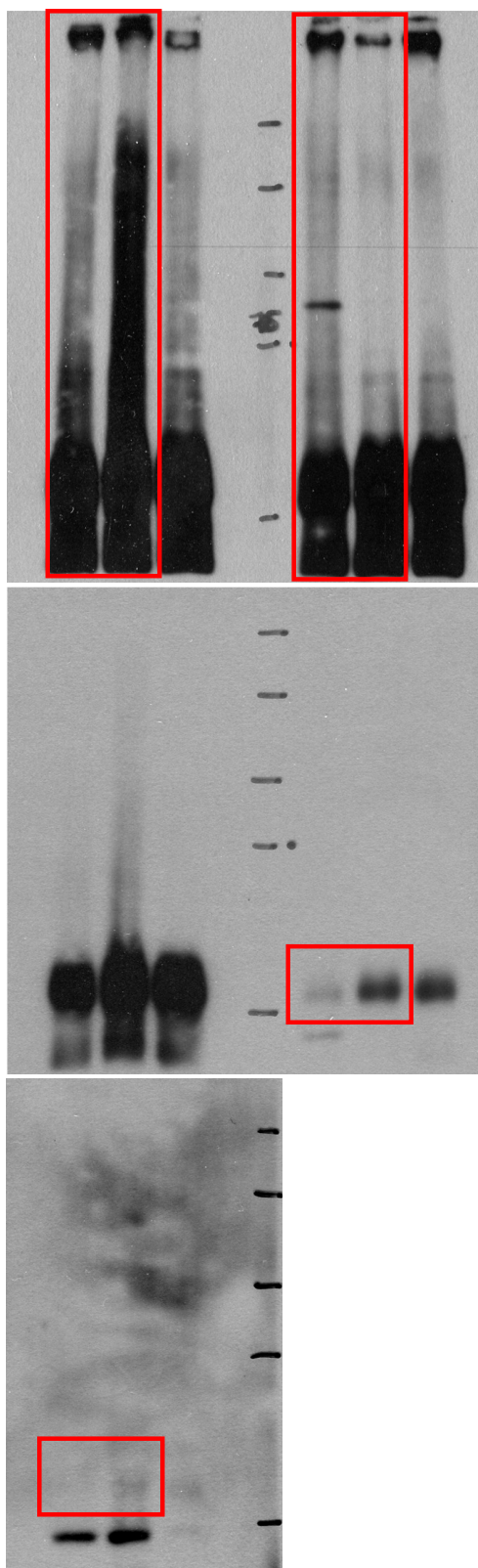

Fig 2

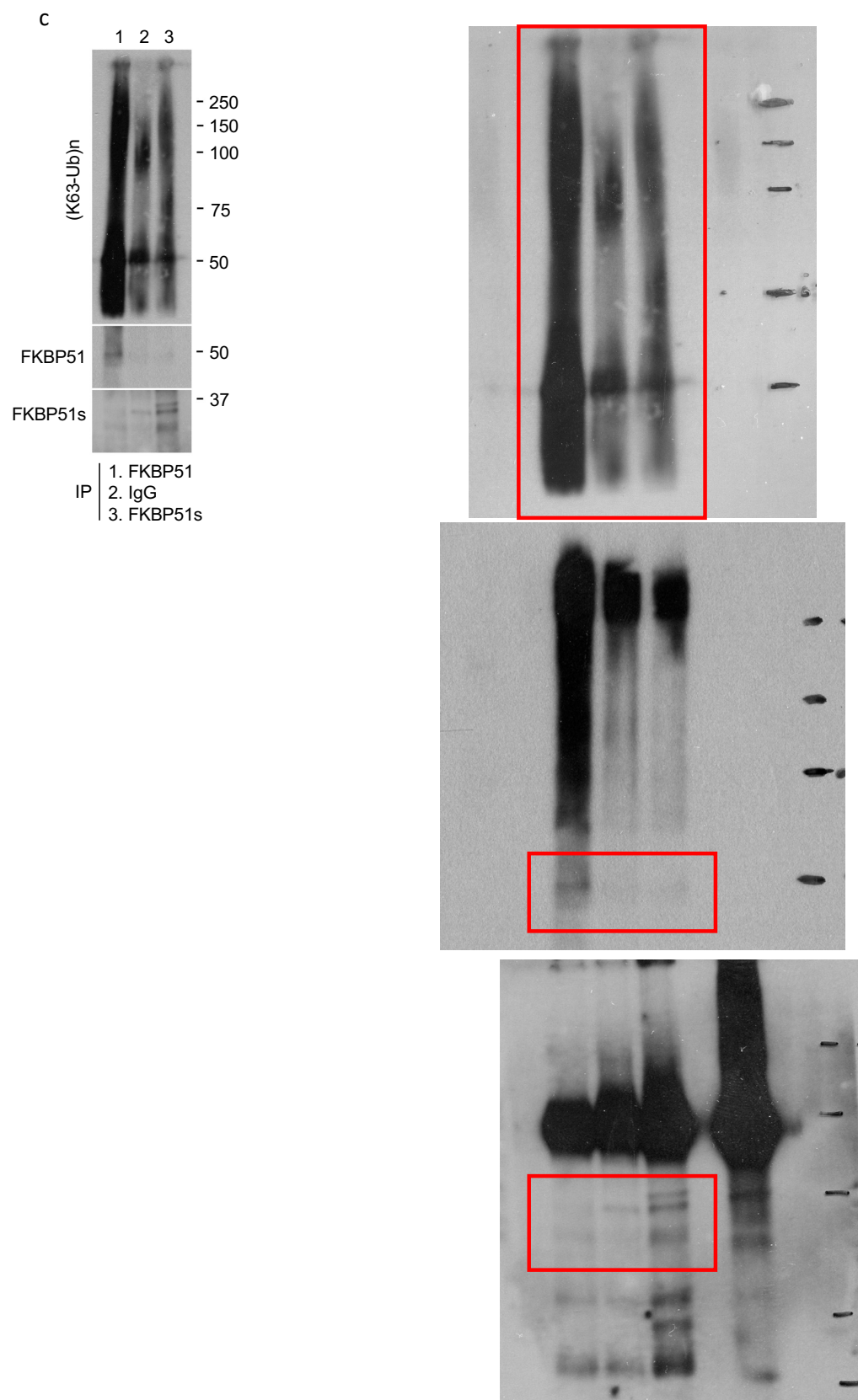

Fig 2

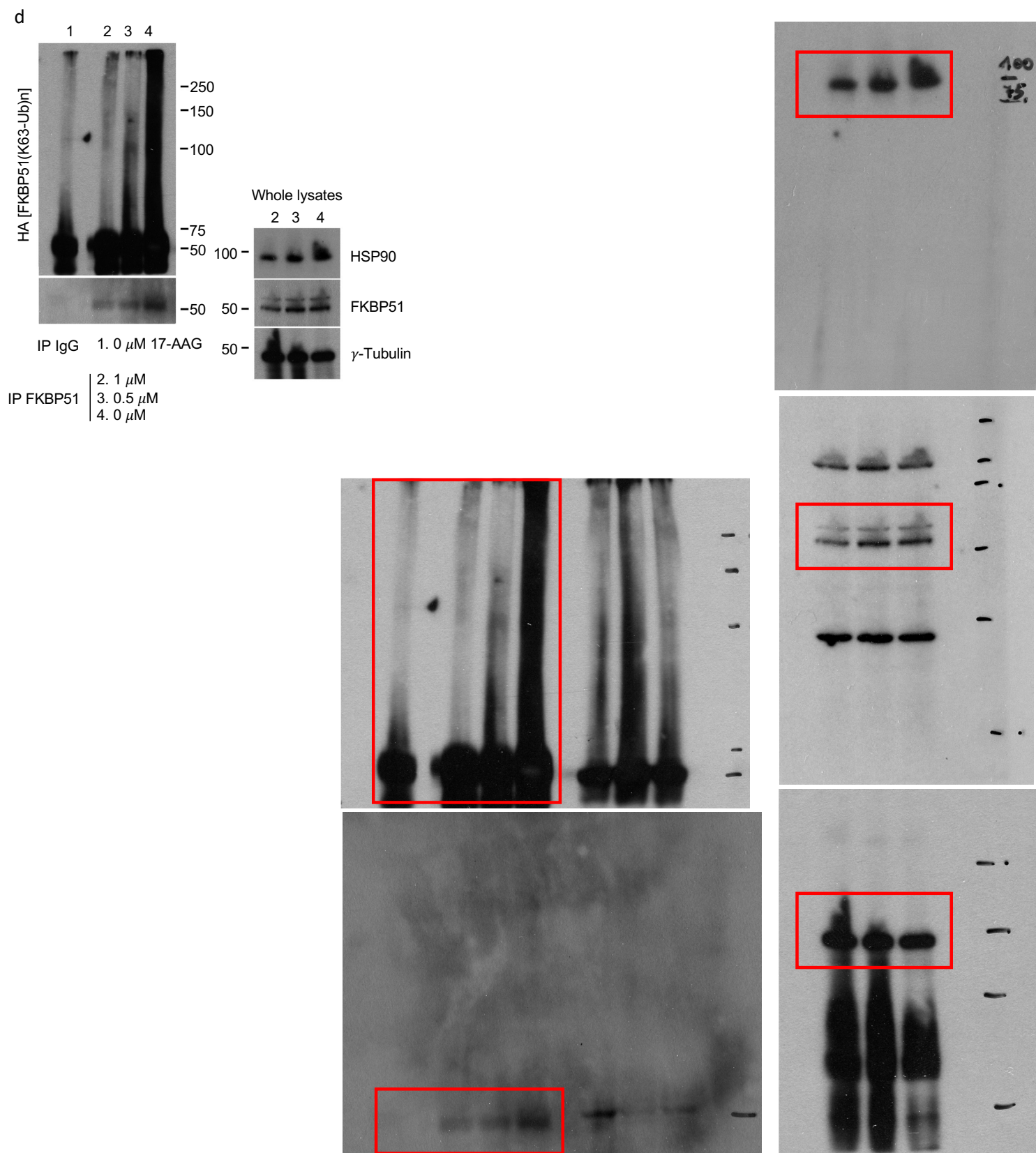

Fig 2

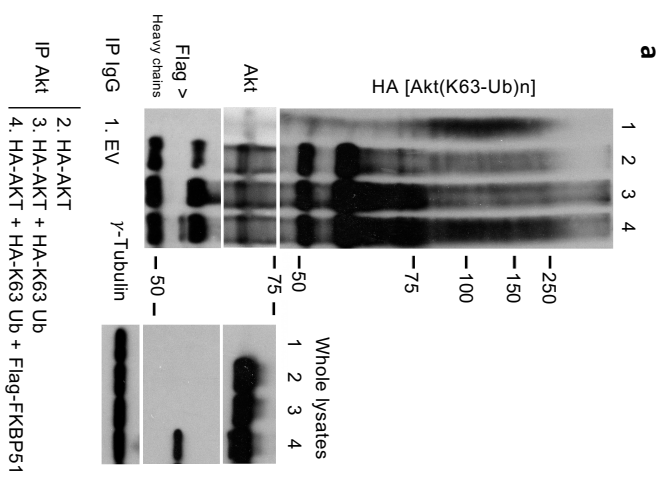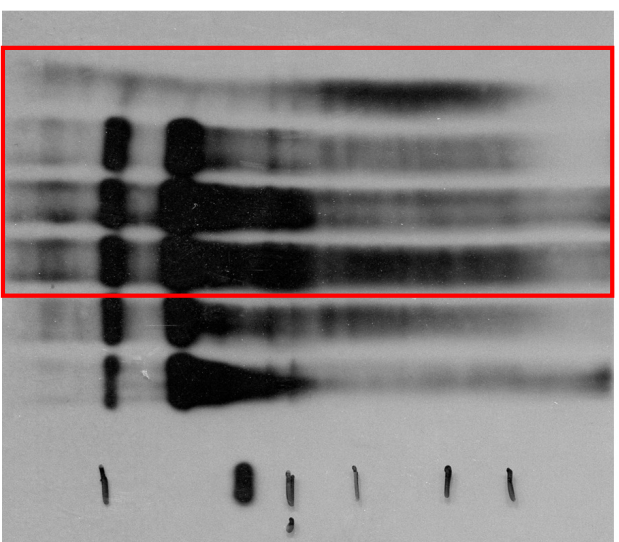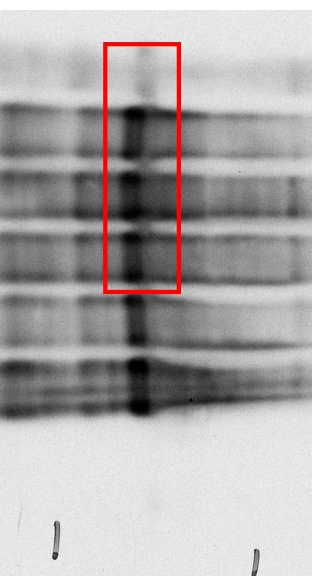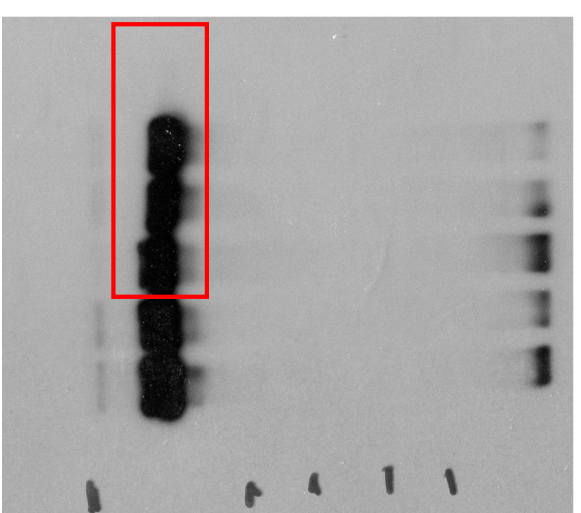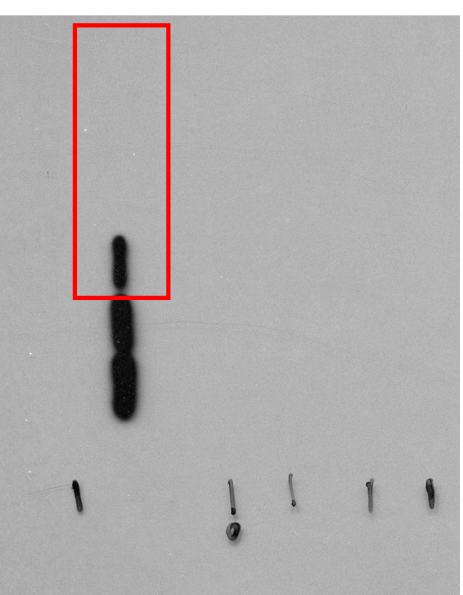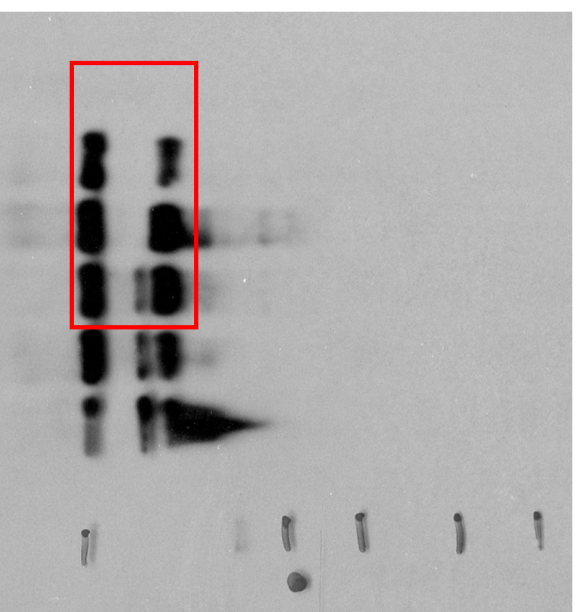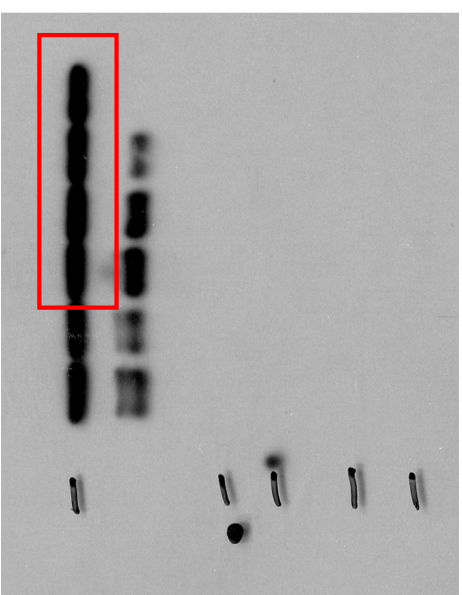

Fig 3

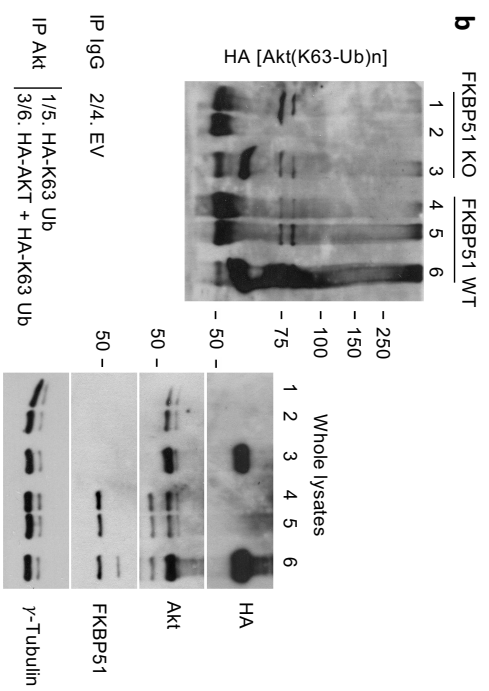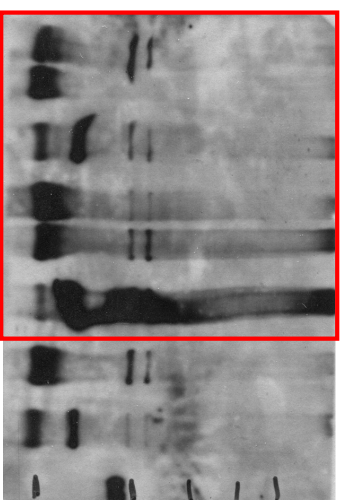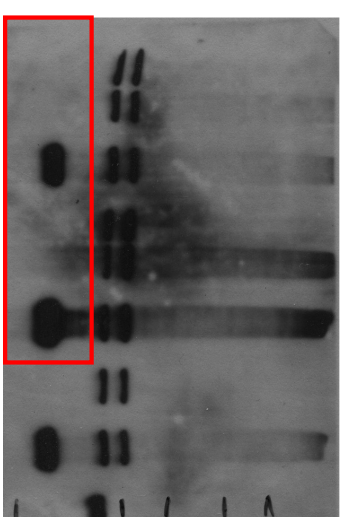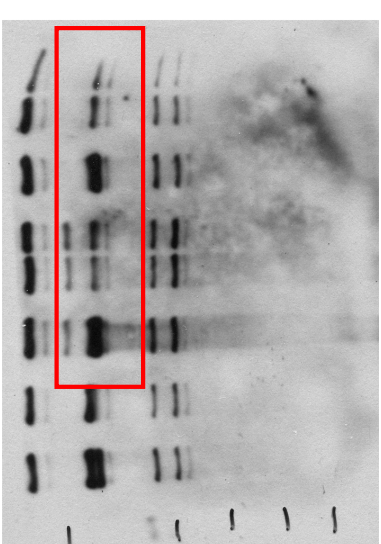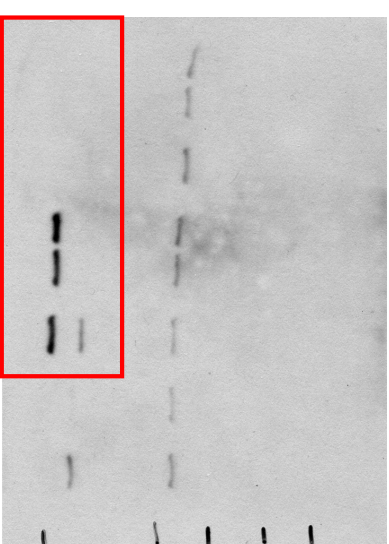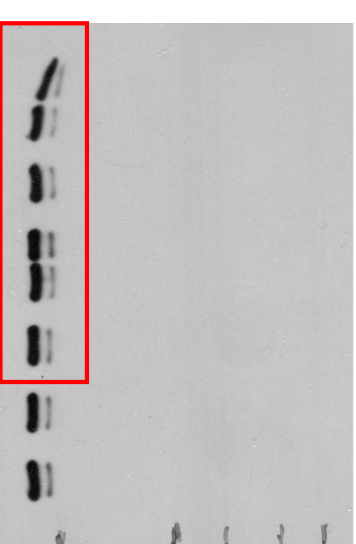

Fig 3

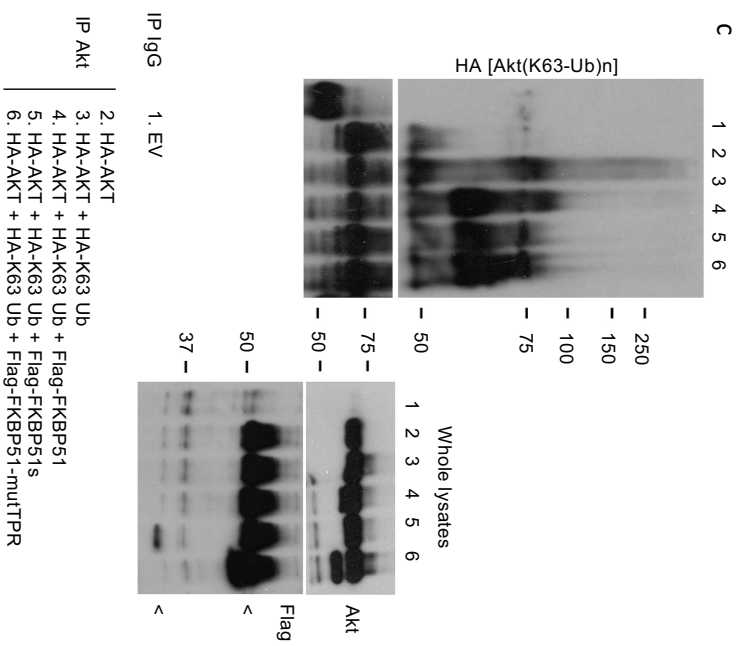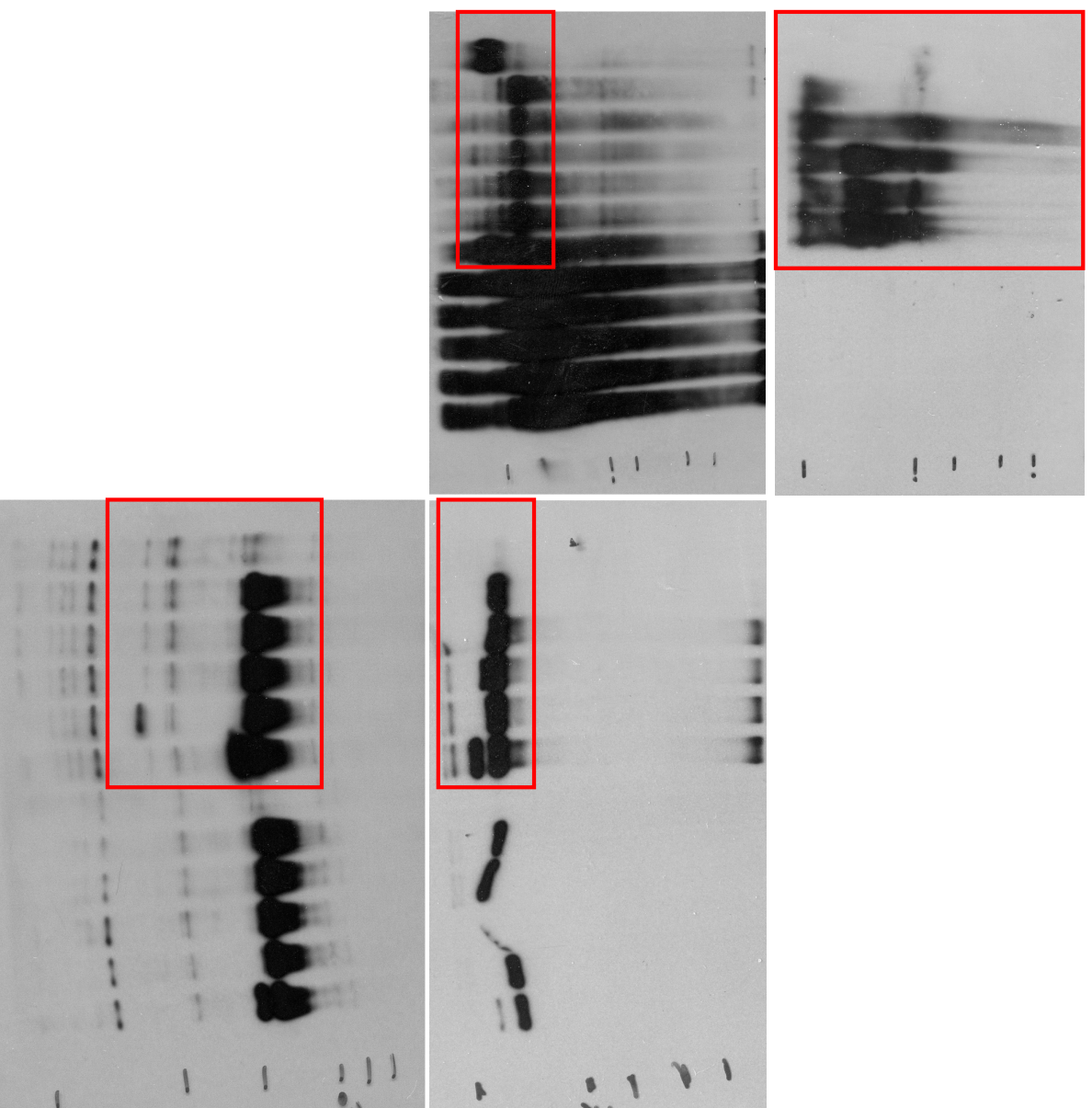

Fig 3

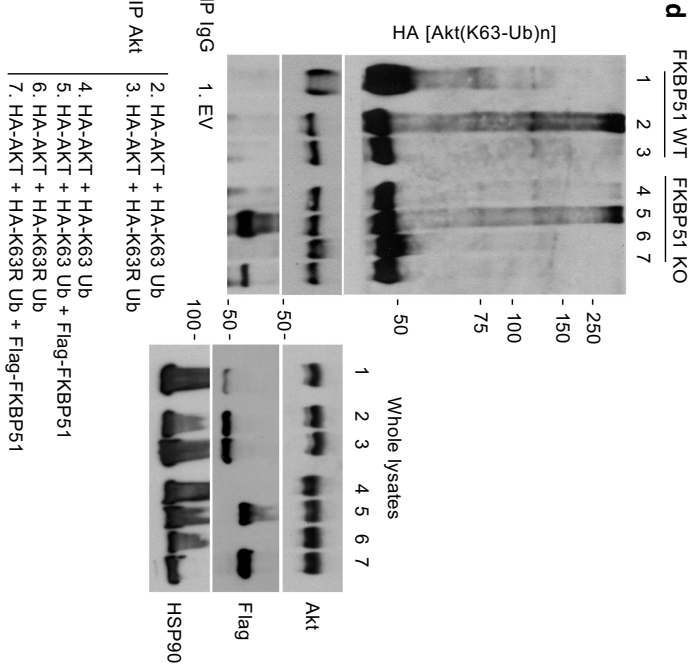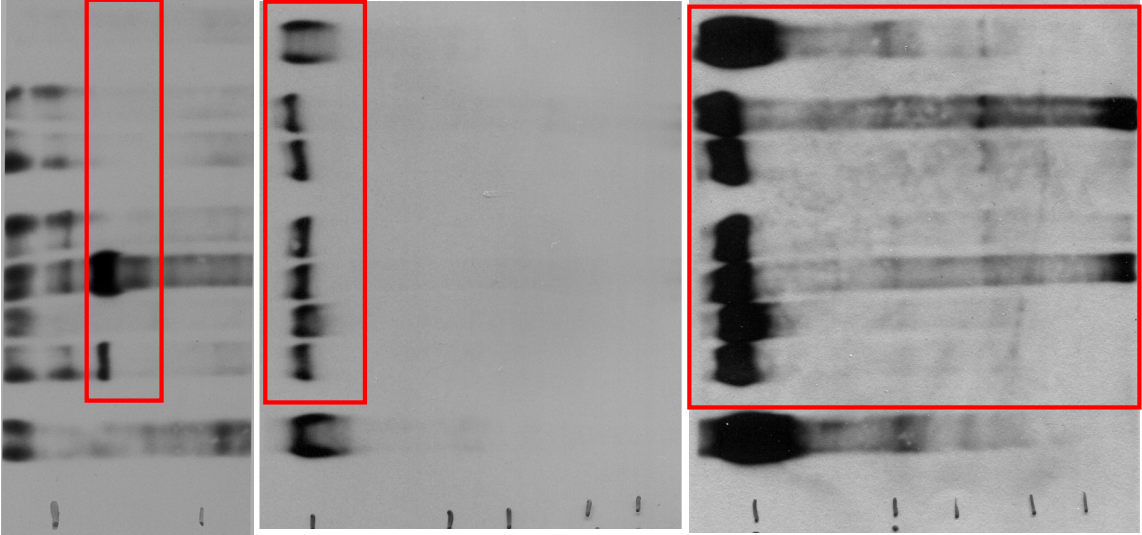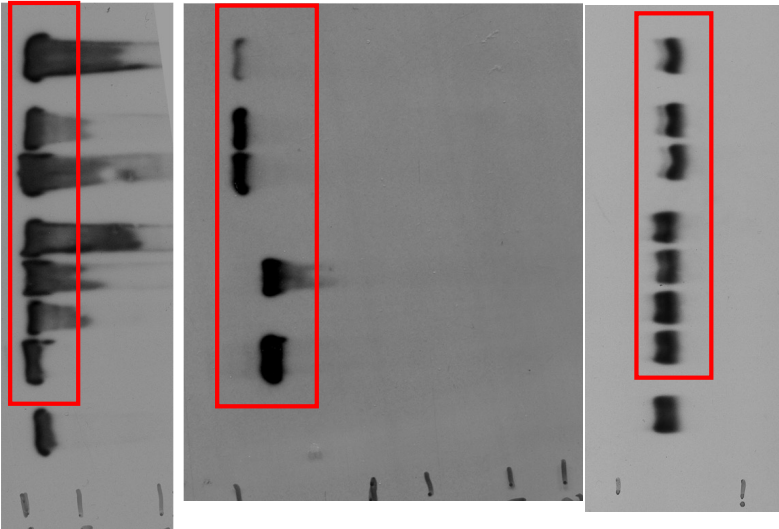

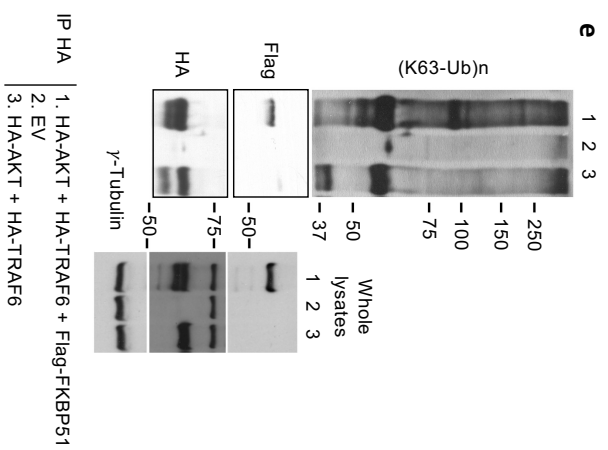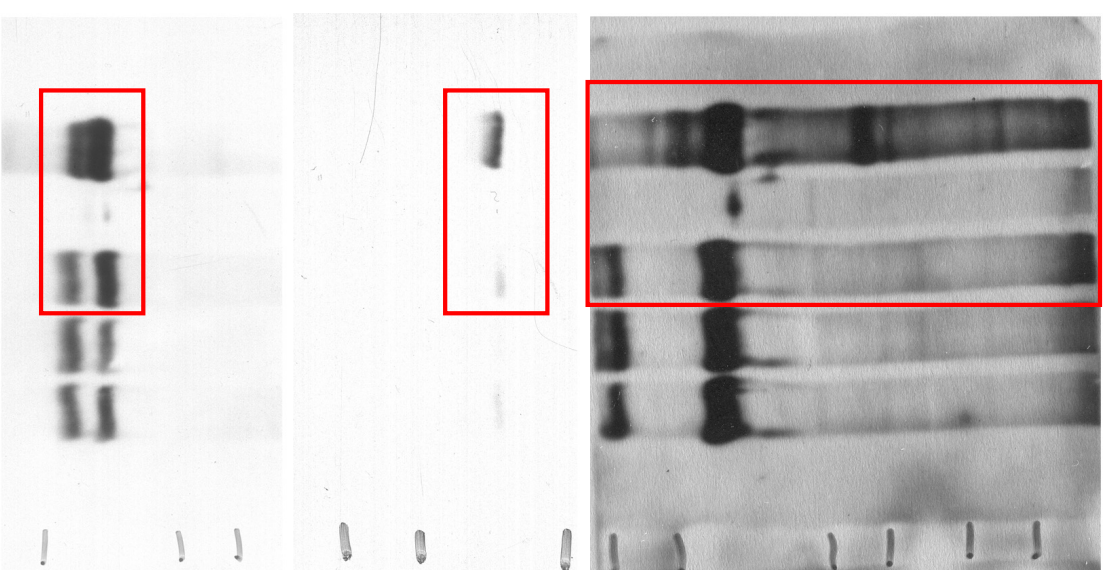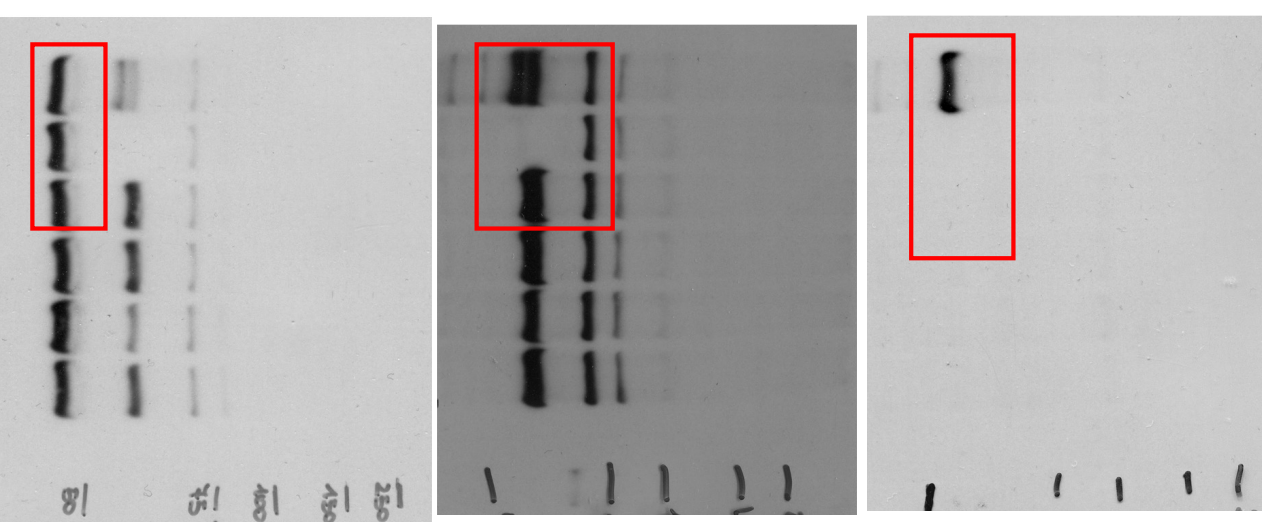

Fig 3

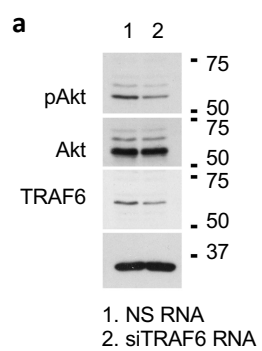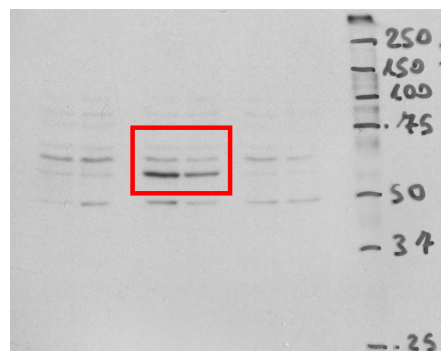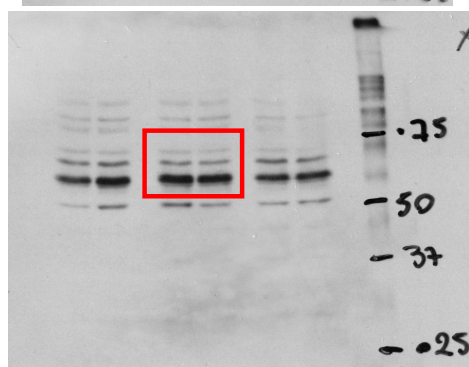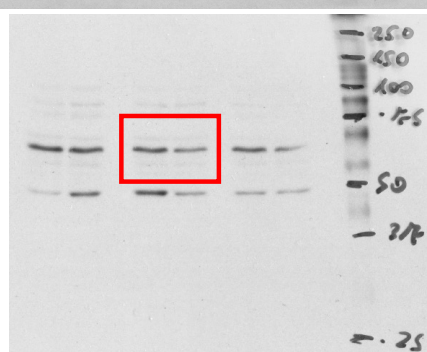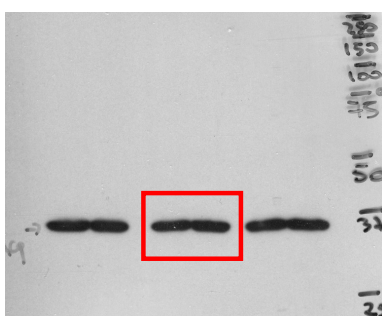

Fig 4

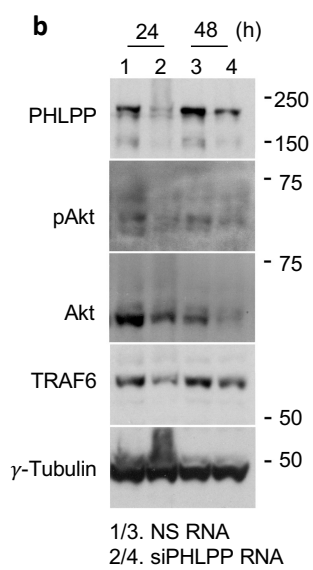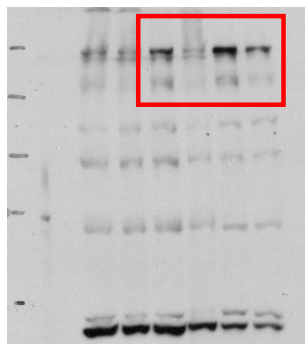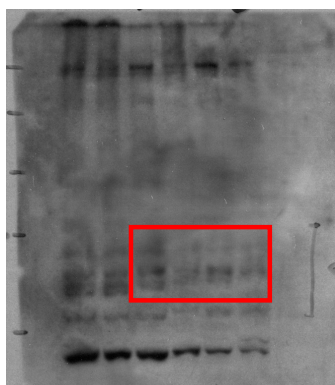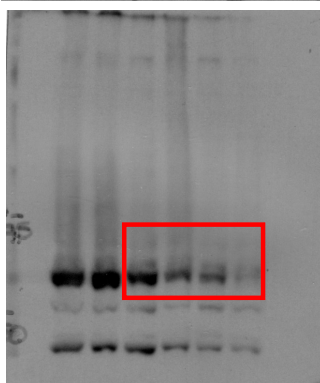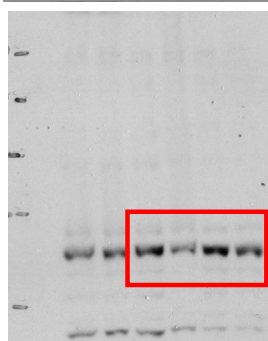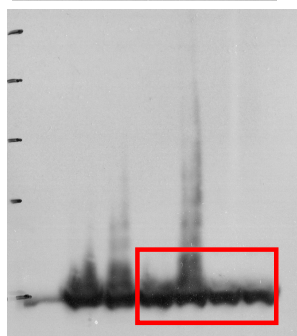

Fig 4

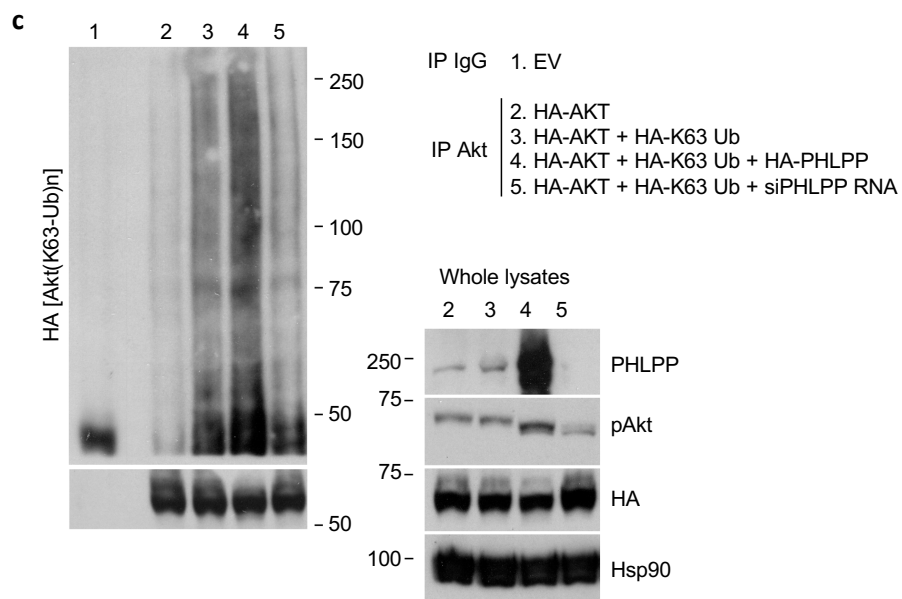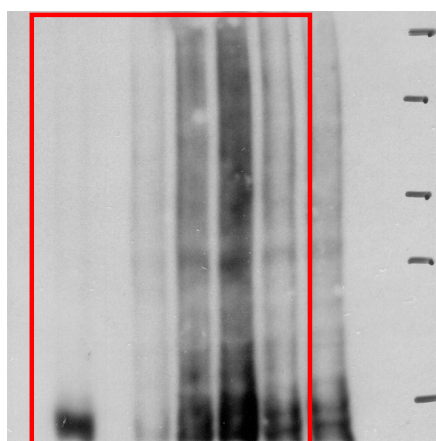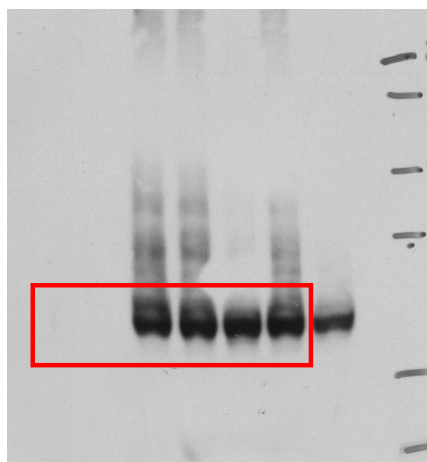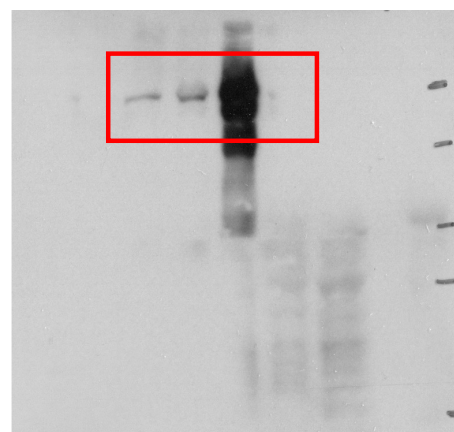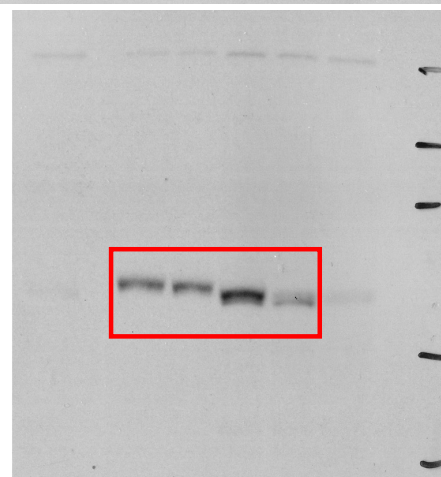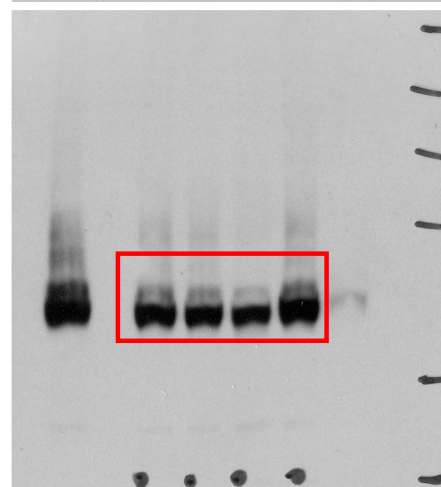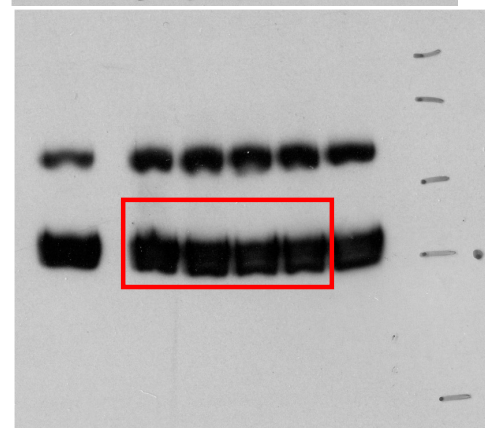

Fig 4

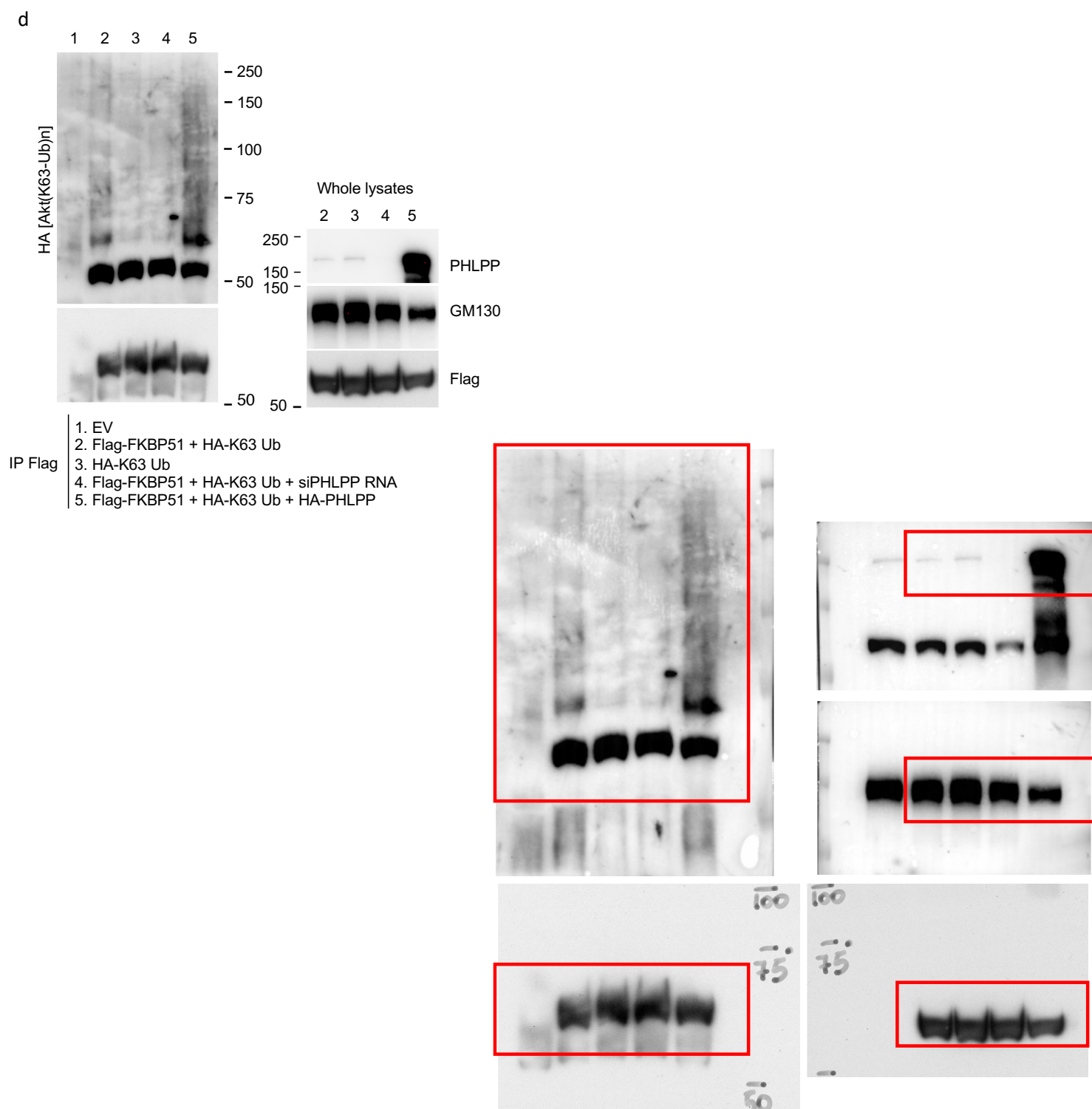

Fig 4

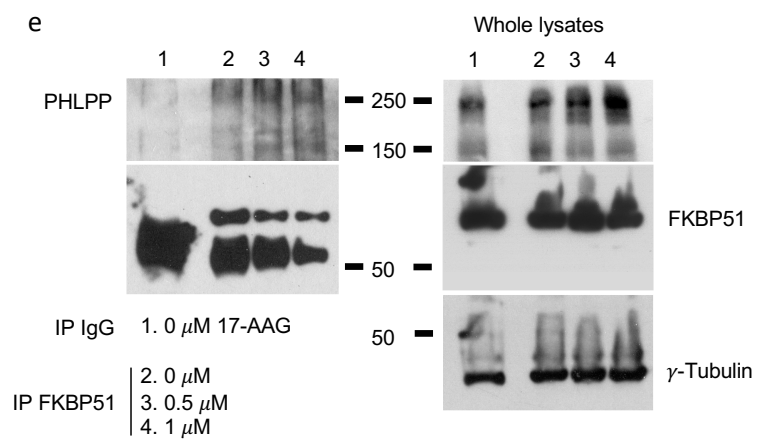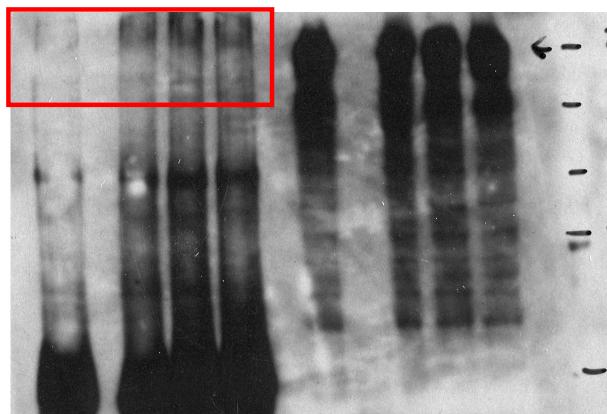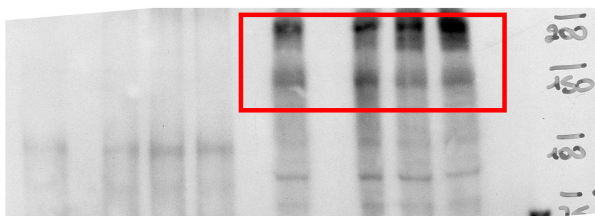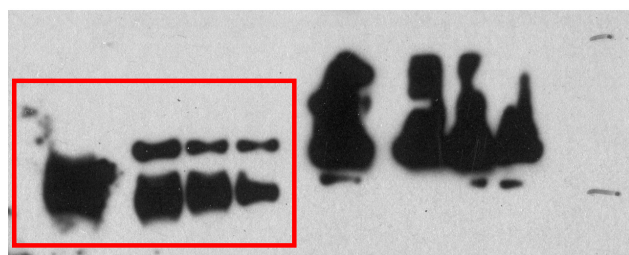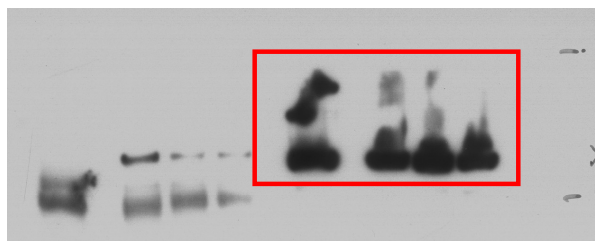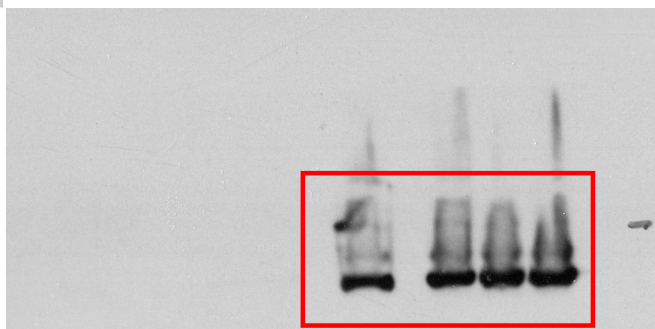

Fig 4

f

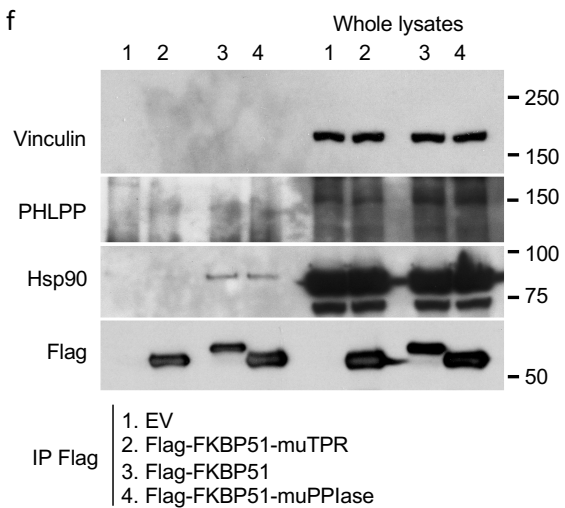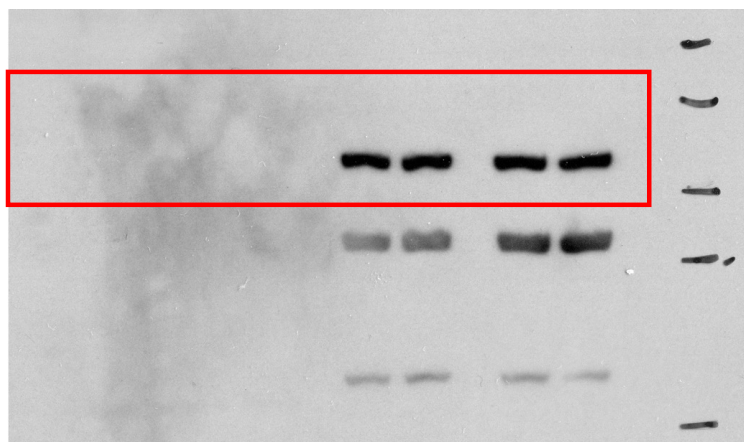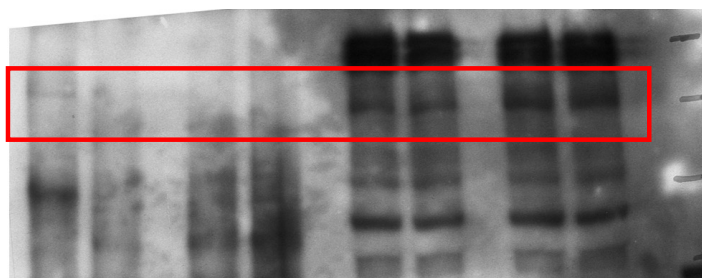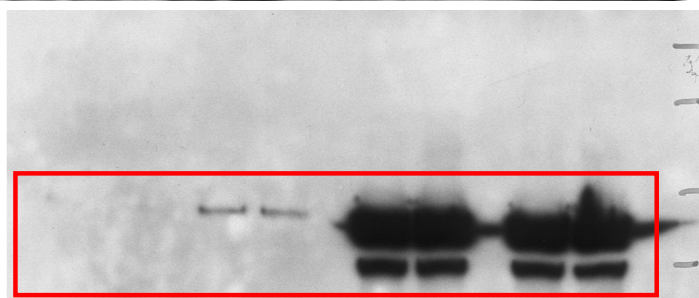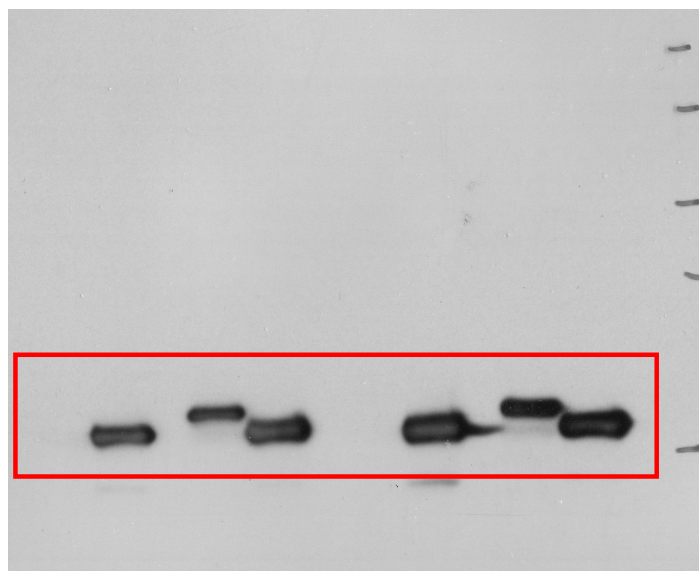

Fig 4

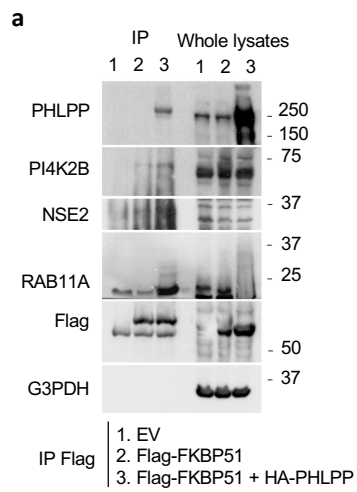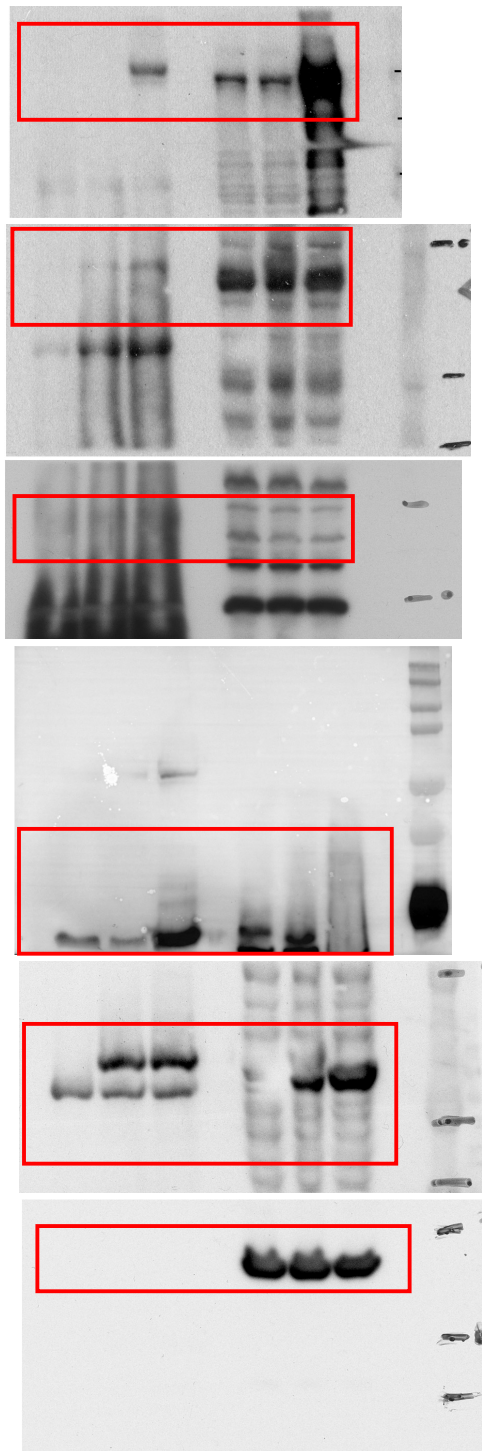

Fig 5

Supplement: Supplementary file 2 — Original Data File [file 41419_2023_5629_MOESM2_ESM.pdf]
